# Safety and immunogenicity of an HIV-1 prefusion-stabilized envelope trimer (Trimer 4571) vaccine in healthy adults: A first-in-human open-label, randomized, dose-escalation, phase 1 clinical trial

**DOI:** 10.1016/j.eclinm.2022.101477

**Published:** 2022-06-01

**Authors:** Katherine V. Houser, Martin R. Gaudinski, Myra Happe, Sandeep Narpala, Raffaello Verardi, Edward K. Sarfo, Angela R. Corrigan, Richard Wu, Ro Shauna Rothwell, Laura Novik, Cynthia S. Hendel, Ingelise J. Gordon, Nina M. Berkowitz, Cora Trelles Cartagena, Alicia T. Widge, Emily E. Coates, Larisa Strom, Somia Hickman, Michelle Conan-Cibotti, Sandra Vazquez, Olga Trofymenko, Sarah Plummer, Judy Stein, Christopher L. Case, Martha Nason, Andrea Biju, Danealle K. Parchment, Anita Changela, Cheng Cheng, Hongying Duan, Hui Geng, I-Ting Teng, Tongqing Zhou, Sarah O'Connell, Chris Barry, Kevin Carlton, Jason G. Gall, Britta Flach, Nicole A. Doria-Rose, Barney S. Graham, Richard A. Koup, Adrian B. McDermott, John R. Mascola, Peter D. Kwong, Julie E. Ledgerwood, VRC 018 Study Team

**Affiliations:** a Vaccine Research Center, National Institute of Allergy and Infectious Diseases, National Institutes of Health, Bethesda, MD, USA; b Commissioned Corps, U.S. Public Health Service, Rockville, MD, USA; c Vaccine Clinical Materials Program, Leidos Biomedical Research, Inc., Frederick National Laboratory for Cancer Research, Frederick, MD, USA; d Biostatistics Research Branch, Division of Clinical Research, National Institute of Allergy and Infectious Diseases, National Institutes of Health, Bethesda, MD, USA

**Keywords:** HIV-1, Vaccine, Trimer 4571, BG505 DS-SOSIP.664, Phase 1 clinical trial, NIH

## Abstract

**Background:**

Advances in therapeutic drugs have increased life-expectancies for HIV-infected individuals, but the need for an effective vaccine remains. We assessed safety and immunogenicity of HIV-1 vaccine, Trimer 4571 (BG505 DS-SOSIP.664) adjuvanted with aluminum hydroxide (alum), in HIV-negative adults.

**Methods:**

We conducted a phase I, randomized, open-label, dose-escalation trial at the National Institutes of Health Clinical Center in Bethesda, MD, USA. Eligible participants were HIV-negative, healthy adults between 18-50 years. Participants were randomized 1:1 to receive Trimer 4571 adjuvanted with 500 mcg alum by either the subcutaneous (SC) or intramuscular (IM) route at weeks 0, 8, and 20 in escalating doses of 100 mcg or 500 mcg. The primary objectives were to evaluate the safety and tolerability of Trimer 4571 with a secondary objective of evaluating vaccine-induced antibody responses. The primary and safety endpoints were evaluated in all participants who received at least one dose of Trimer 4571. Trial results were summarized using descriptive statistics. This trial is registered at ClinicalTrials.gov, NCT03783130.

**Findings:**

Between March 7 and September 11, 2019, 16 HIV-negative participants were enrolled, including six (38%) males and ten (62%) females. All participants received three doses of Trimer 4571. Solicited reactogenicity was mild to moderate in severity, with one isolated instance of severe injection site redness (6%) following a third 500 mcg SC administration. The most commonly reported solicited symptoms included mild injection site tenderness in 14 (88%) and mild myalgia in six (38%) participants. The most frequent unsolicited adverse event attributed to vaccination was mild injection site pruritus in six (38%) participants. Vaccine-induced seropositivity occurred in seven (44%) participants and resolved in all but one (6%). No serious adverse events occurred. Trimer 4571-specific binding antibodies were detected in all groups two weeks after regimen completion, primarily focused on the glycan-free trimer base. Neutralizing antibody activity was limited to the 500 mcg dose groups.

**Interpretation:**

Trimer 4571 was safe, well tolerated, and immunogenic in this first-in-human trial. While this phase 1 trial is limited in size, our results inform and support further evaluation of prefusion-stabilized HIV-1 envelope trimers as a component of vaccine design strategies to generate broadly neutralizing antibodies against HIV-1.

**Funding:**

Intramural Research Program of the Vaccine Research Center, National Institute of Allergy and Infectious Diseases, National Institutes of Health.

**Research in context**

**Evidence before this study**

We searched PubMed from database inception up to August 23, 2021, using the terms “HIV-1” AND “vaccine” AND “clinical trials” AND “Env trimer.” We found no publications from clinical trials involving the administration of a soluble, structurally stabilized HIV-1 envelope (Env) glycoprotein trimer (Trimer 4571) in healthy HIV-negative adults. The goal of our first-in-human phase 1 trial was to evaluate the safety and immunogenicity of Trimer 4571 adjuvanted with aluminum hydroxide (alum) in healthy HIV-negative adults.

**Added value of this study**

Trimer 4571 was the first prefusion-closed stabilized HIV-1 Env trimer to be evaluated in humans and was safe and well tolerated when adjuvanted with alum and administered by subcutaneous and intramuscular routes. Trimer 4571-specific antibodies, primarily focused on the glycan-free trimer base, were identified two weeks after regimen completion, with no detectable responses to CD4-induced epitopes. Limited neutralization was detected with a significant portion of vaccine-induced antibodies targeting the glycan-free base of the trimer.

**Implications of all the available evidence**

The results of this first-in-human trial of a prefusion-closed stabilized HIV-1 Env trimer informs ongoing vaccine efforts to define the human immune responses capable of producing broadly neutralizing antibodies. Future studies will explore Trimer 4571 in various prime-boost vaccine regimens, with more potent adjuvants, and with additional structural modifications including masking of the glycan-free trimer base to improve the neutralization breadth and potency of vaccine-induced antibodies.

Alt-text: Unlabelled box

## Introduction

The HIV-1 epidemic persists despite tremendous gains in understanding HIV-1 biology in addition to the use of antiretroviral medications to treat and prevent infections.^1^ As of 2020, 38 million people globally were living with HIV-1 with approximately 1·7 million new infections occurring annually.^2^ Despite decades of research, only seven late phase 2b and phase 3 HIV-1 vaccine clinical trials have occurred, with the RV144 trial (NCT00223080) resulting in the highest measurable vaccine efficacy of 31·2% after three years.^3^ This highlights the need for an effective HIV-1 vaccine capable of eliciting broadly neutralizing antibodies (bnAbs) to prevent infection from the diverse set of natural viral isolates. Induction of bnAbs through active immunization is a main goal of an effective HIV vaccine but will require innovative concepts for both vaccine regimen and immunogen design.^4^

The HIV-1 envelope (Env) glycoprotein trimer, composed of gp120 and gp41 heterodimers, is essential for mediating viral entry into the host cell and is the principal antibody target for HIV-1.^4^ The first efficacy trials of HIV-1 vaccines designed to induce humoral protection (VAX 004, NCT00002441; VAX 003, NCT00006327) evaluated monomeric recombinant Env gp120 subunit vaccines.^5^^,^^6^ While these vaccines were safe and elicited neutralizing antibodies in clinical trials,^7^ the vaccine-induced antibody responses did not prevent HIV-1 infection.^8^ The failure of these vaccines in eliciting protective bnAbs can be attributed to a myriad of HIV-1 immune evasion strategies, including antigenic diversification during replication and the dense shield of surface glycans on Env preventing exposure of critical antigenic epitopes to the immune system.^4^^,^^9^ The Env trimer is moreover structurally dynamic, and its various conformations elicit distinct antibody responses.^10^, ^11^, ^12^ The closed prefusion conformation is recognized by potent bnAbs, whereas antibodies targeting regions exposed in the open conformation induced by CD4 binding (CD4-induced epitopes) are weakly or non-neutralizing and ineffective at preventing infection.^13^^,^^14^

Structure-based vaccine design has led to stabilized class 1 fusion protein viral immunogens that are locked in the prefusion-closed conformation capable of eliciting protective bnAbs.^15^ This approach has recently been successfully utilized to create a protein subunit vaccine against respiratory syncytial virus as well as mRNA vaccines encoding SARS-CoV-2 spike proteins.^16^^,^^17^ For HIV-1, a soluble protein trimer immunogen was designed based on the clade A strain BG505 (BG505 SOSIP.664).^14^^,^^18^ Prior studies involving the BG505 SOSIP.664 trimer indicated that although this construct contained stabilizing mutations, it could still be recognized by non-neutralizing, CD4-induced antibodies.^12^ Therefore, we introduced an additional 201C-433C disulfide mutation (DS) within gp120 to prevent any CD4-induced conformational change.^12^ This modified soluble, prefusion-closed conformation immunogen, Trimer 4571 (BG505 DS-SOSIP.664), exhibited the desired antigenic profile and was resistant to CD4-induced conformational change.^12^ In preclinical studies, it was found to be safe and resulted in neutralizing antibodies in both guinea pigs and rhesus macaques when administered with an adjuvant.^19^, ^20^, ^21^ Furthermore, when used in combination vaccine regimens with a HIV-1 fusion peptide-coupled carrier, Trimer 4571 was shown to result in cross-clade neutralizing antibodies in mice, guinea pigs, and rhesus macaques.^22^

When evaluating vaccination regimens involving multiple immunogens administered either simultaneously or in a vaccination series, investigating a component individually first allows us to discern its distinct safety and immunogenicity profile. Therefore, prior to using Trimer 4571 in combination with other immunogens we evaluated it separately in this phase 1 trial of healthy, HIV-negative adults. Trimer 4571 was administered with alum at either 100 mcg or 500 mcg doses in a three-injection regimen (weeks 0, 8, and 20) by either intramuscular (IM) or subcutaneous (SC) routes. Herein we report the safety and vaccine-induced antibody response results from this first-in-human phase 1 clinical trial.

## Methods

### Study design and participants

This study (ClinicalTrials.gov NCT03783130) was a single site, open-label, dose-escalation, randomized phase 1 clinical trial examining the safety and immunogenicity of Trimer 4571 adjuvanted with aluminum hydroxide suspension (alum) in a three-injection regimen administered either IM or SC. The trial was conducted and sponsored by the Vaccine Research Center (VRC) Clinical Trials Program (CTP), National Institute of Allergy and Infectious Diseases (NIAID) at the National Institutes of Health (NIH) Clinical Center, Bethesda, MD, USA. The trial protocol was approved by NIAID Institutional Review Board (IRB). Study participants were recruited through IRB-approved advertising from the greater Washington, DC area. CONSORT reporting guidelines were adhered to throughout the trial.

Eligible participants were healthy, HIV-negative adults between 18 and 50 years of age. Inclusion criteria included good general health demonstrated through physical examination and laboratory tests. Exclusion criteria included previous receipt of an investigational HIV vaccine prior to enrollment. A full list of inclusion and exclusion criteria is included in the trial protocol. All participants provided written informed consent prior to enrollment.

### Randomization and masking

Eligible participants were randomized 1:1 into the IM or SC 100 mcg dose groups. Subsequent participants were enrolled and randomized 1:1 into the IM or SC 500 mcg dose groups once the criteria for dose-escalation were met and interim safety reviews were completed. Block randomization was done using R software (Version 4·1), using block sizes of 4 and 6. Vaccines were administered open label.

### Products and procedures

Trimer 4571 (VRC-HIVRGP096-00VP) was produced in a Chinese Hamster Ovary (CHO) DG44 cell line in accordance with Good Manufacturing Practices (cGMP) regulations at the VRC Pilot Plant operated by the Vaccine Clinical Materials Program (VCMP), Leidos Biomedical Research, Inc., Frederick, MD.^23^ Trimer 4571 was derived from the clade A strain BG505 and built to mimic the native HIV-1 Env trimer in a stabilized prefusion closed conformational state. The soluble, stabilized conformation of Trimer 4571 was achieved by stabilizing modifications and engineered disulfide bonds, the truncation of gp41 at residue 664, and the addition of a 201C-433C disulfide mutation within gp120.^10^^,^^12^^,^^14^

Participants received Trimer 4571 by needle and syringe in a 1 mL volume per injection into either the deltoid muscle or subcutaneous tissue in the posterior upper arm. Participants received either a 100 mcg or 500 mcg dose adjuvanted with 500 mcg alum at weeks 0, 8, and 20. All administrations were monitored by a study clinician. Participants were observed for a minimum of 30 minutes after each injection and the injection site was inspected. Local and systemic reactogenicity were solicited from participants for seven days after each vaccination. Unsolicited adverse events (AEs) were recorded for 28 days after each vaccination, while severe adverse events (SAEs) and new chronic medical conditions were recorded through the duration of the study. Adverse events were coded in accordance with the Medical Dictionary for Regulatory Activities and graded for severity based on the Division of AIDS (DAIDS) Table for Grading the Severity of Adult and Pediatric Adverse Events, Version 2·1. Participants were followed for 40 weeks after the first study injection.

All participants were monitored for vaccine-induced seropositivity (VISP) during the trial. VISP can occur in the absence of HIV infection when vaccine-induced antibodies are detected by standard clinical HIV screening assays (Orthos VITROS Immunodiagnostic Products HIV Combo Reagent Pack, VITROS 3600 system).^24^ Participants with VISP were confirmed to be HIV-negative by PCR, counseled, and provided letters explaining the VISP results.

Serum samples were collected at protocol-specified timepoints for immunogenicity analysis of vaccine-induced antibody responses. The immunogenicity assays, including MSD electrochemiluminescence immunoassays (ECLIAs), ELISAs, pseudoviral neutralization assays, and electron microscopy polyclonal epitope mapping (EMPEM), are described in Supplementary material 1.

### Outcomes

The primary outcome of the study evaluated the safety and tolerability of alum-adjuvanted Trimer 4571 administered at either 100 mcg or 500 mcg by IM and SC routes. The secondary outcome investigated the vaccine-induced antibody response two weeks after regimen completion.

### Statistical analysis

All participants completed their vaccination schedules and were analyzed for safety, reactogenicity, and vaccine-induced antibody responses. Sample size calculations for safety were expressed in terms of the ability to detect SAEs. Sample sizes were chosen so that across the trial groups there was a 97% chance to observe at least one SAE if the true rate was at least 0·30, and 85% chance to observe no SAE if the true rate was less than 0·01. Adjustments for multiple comparisons were not performed. All immunological data were log-transformed and comparisons were made between group geometric means using Welch's t-test. Comparisons between timepoints or changes over time between groups were analyzed using individual differences of the log-transformed data. Statistical analyses were performed with R, Version (4·1).

### Role of funding source

The funder of the study had a role in study design, data collection, data analysis, data interpretation, and writing of this report. The corresponding author had full access to all the data in the study and had final responsibility to submit for publication.

## Results

### Clinical trial design and participants

**Figure 1:**
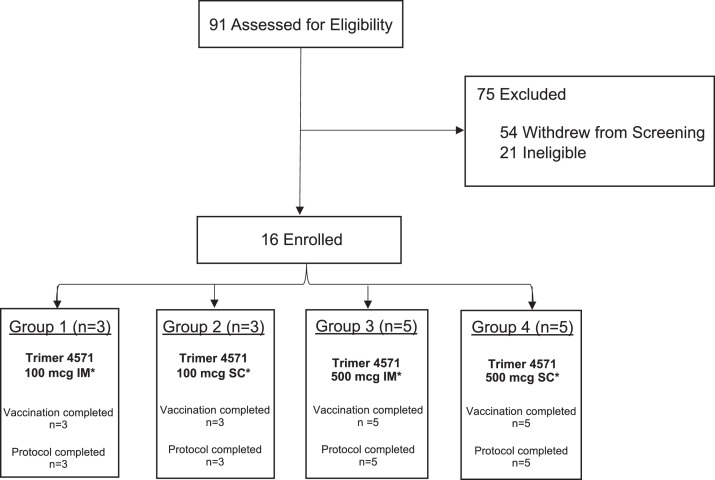
Trial CONSORT diagram. Participants were enrolled according to dose-escalation protocol and randomized to receive three doses of adjuvanted Trimer 4571 at weeks 0, 8, and 20. All participants completed the protocol. IM denotes intramuscular, and SC subcutaneous route of vaccine administration. Participants who withdrew during enrollment did so because: time commitment for study procedures (10 participants, 19%), concern of potential risks of study procedures (28 participants, 52%), number of procedures/blood draws (2 participants, 4%), lost contact (5 participants, 9%), study closed to enrollment (7 participants, 13%), participants chose another study (1 participants, 2%) and other (29, 54%). Of the twenty-eight (28) participants that withdrew due to concern of potential risks, 23 (82%) had concerns with vaccine induced seropositivity (VISP). Participants could choose more than one withdrawal reason/category. * 500 mcg of alum was field mixed with Trimer 4571 for all study groups.

The trial enrolled 16 HIV-negative participants between March 7 and September 11, 2019. The participants included six (38%) males and ten (62%) females, with a mean (±SD) age of 33 (±10) years (Table 1). All participants completed the protocol and received all scheduled vaccinations, for a total of 48 Trimer 4571 administrations (Figure 1). In-person clinic follow-up visits were interrupted beginning March 13, 2020, due to the COVID-19 pandemic; instead, the remaining visits were conducted by phone. This impacted a total of eight participants from the 500 mcg dose groups, resulting in missed sample collections during the final study visits (Table S1).

**Table 1:** Baseline demographic characteristics of the participants.

Category	Group 1 100 mcg IM	Group 2 100 mcg SC	Group 3 500 mcg IM	Group 4 500 mcg SC	Overall
	(N=3)	(N=3)	(N=5)	(N=5)	(N=16)
**Sex**					
Male	1 (33·3%)	1 (33·3%)	2 (40·0%)	2 (40·0%)	6 (37·5%)
Female	2 (66·7%)	2 (66·7%)	3 (60·0%)	3 (60·0%)	10 (62·5%)
**Age**					
Mean (SD)	32·3 (12·7)	35·7 (12·5)	32·4 (8·1)	32·4 (10·9)	33·0 (9·7)
Range	[24·0, 47·0]	[23·0, 48·0]	[23·0, 41·0]	[22·0, 44·0]	[22·0, 48·0]
**Race**					
Asian	0 (0·0%)	0 (0·0%)	0 (0·0%)	2 (40·0%)	2 (12·5%)
Black or African American	1 (33·3%)	0 (0·0%)	0 (0·0%)	1 (20·0%)	2 (12·5%)
White	1 (33·3%)	3(100·0%)	4 (80·0%)	2 (40·0%)	10 (62·5%)
Multiracial	1 (33·3%)	0 (0·0%)	1 (20·0%)	0 (0·0%)	2 (12·5%)
**Ethnicity**					
Non-Hispanic/Latino	2 (66·7%)	3 (100·0%)	5 (100·0%)	5 (100·0%)	15 (93·8%)
Hispanic/Latino	1 (33·3%)	0 (0·0%)	0 (0·0%)	0 (0·0%)	1 (6·3%)
**BMI**					
Mean (SD)	29·4 (5·0)	27·4 (4·3)	23·4 (3·7)	26·0 (5·7)	26·1 (4·8)
Range	[25·2, 35·0]	[22·5, 30·1]	[17·8, 26·9]	[19·1, 32·5]	[17·8, 35·0]
**Education**					
High school graduate/GED	0 (0·0%)	0 (0·0%)	1 (20·0%)	0 (0·0%)	1 (6·3%)
College/University	2 (66·7%)	1 (33·3%)	3 (60·0%)	3 (60·0%)	9 (56·3%)
Advanced degree	1 (33·3%)	2 (66·7%)	1 (20·0%)	2 (40·0%)	6 (37·5%)

Sex, race and ethnic group were reported by the participants. Data are n (%) or mean (SD) unless otherwise specified. Sex was reported based on the sex assigned at birth. Body mass index (BMI) represents BMI at enrollment and is reported as the weight in kilograms divided by the square of the height in meters.

### Safety

**Figure 2:**
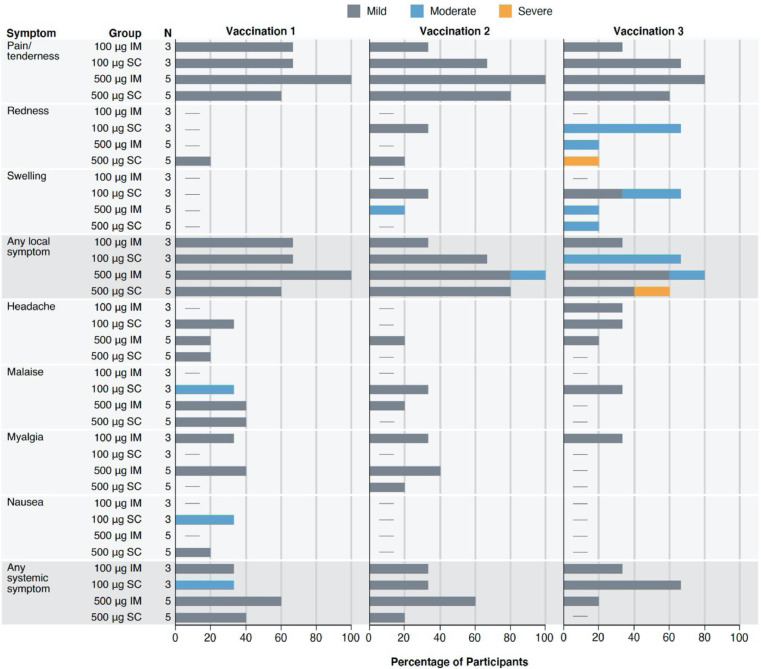
Maximum local and systemic reactogenicity reported within 7 days after receipt of each Trimer 4571 vaccination according to dose and route of administration. Percent of participants who reported a local or systemic symptom (y-axis) in the seven days following each vaccination. The severity of solicited adverse events was graded mild, moderate, and severe. Data are n (%). IM denotes intramuscular, and SC subcutaneous.

Solicited local and systemic reactogenicity following vaccination were mild to moderate, apart from one report of severe injection site redness following a third 500 mcg SC administration (Figure 2, Table S2). The redness peaked at 16 cm and resolved by day six without sequelae. The most commonly reported solicited symptoms included mild injection site tenderness in 14 (88%) and mild myalgia in six (38%) participants. A total of ten unsolicited AEs were attributed to vaccination (Table S3). Nine of these events involved mild pruritis at the injection site, with one additional report of mild neutropenia seven days after the second product administration. All AEs resolved without sequelae. No serious adverse events occurred.

Vaccine-induced seropositivity (VISP), where vaccine-induced antibodies are detected by standard HIV clinical screening assays despite the absence of infection, occurred in seven participants during the trial.^24^ Participants were confirmed to be HIV negative by PCR in each instance. Six (86%) of these participants were in the 500 mcg dose groups, with four (57%) receiving Trimer 4571 by IM administration. VISP appeared only after sequential vaccinations and resolved in all but one participant by the end of the trial. We were unable to confirm resolution in the last participant due to missed in-person visits during the COVID-19 pandemic, after which the participant moved from the area and was unavailable for follow up.

### Trimer 4571-induced antibody responses

**Figure 3:**
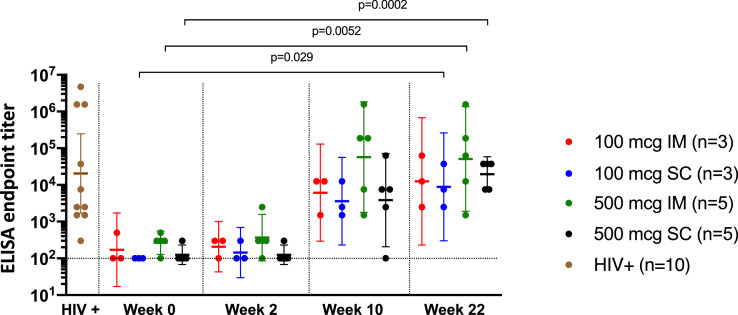
Vaccination with Trimer 4571 induces trimer-specific antibody responses by ELISA. Trimer 4571-specific antibody titers in serum were measured by ELISA at baseline and two weeks after each vaccination. HIV+ samples were used as positive controls. ELISA endpoint titer was defined as the reciprocal of the greatest dilution with an optical density value above 0·1. Geometric mean titers (GMTs) with 95% confidence intervals (CIs) are indicated by error bars. Horizontal dotted line indicates the assay negative cut off. IM denotes intramuscular, and SC subcutaneous. Significant differences from baseline to two weeks after regimen completion were observed for 100 mcg SC (p=0·029), 500 mcg IM (p=0·0052), and 500 mcg SC (p=0·0002) groups.

To assess vaccine-induced antibody responses throughout the trial, we utilized a lectin-capture method to coat ELISA plates with Trimer 4571. As expected, we did not detect Trimer 4571-specific antibodies at baseline (week 0) (Figure 3). Participants in all groups displayed detectable Trimer 4571-specific antibodies two weeks following the second vaccination (week 10). We observed the highest antibody responses in the 500 mcg IM group two weeks after regimen completion (week 22) with a geometric mean ELISA endpoint titer of 50950 (95% CI 1923-1349873). In comparison, the 100 mcg IM, 100 mcg SC, and 500 mcg SC geometric mean ELISA endpoint titers were 12500 (95% CI 229-681158), 8892 (95 % CI 302-262080), and 19699 (95% CI 6593-58858), respectively. These geometric mean endpoint titers were significantly higher than baseline levels for the 100 mcg (p=0·029) and 500 mcg (p=0·0002) SC groups and the 500 mcg IM group (p=0·0052). Durability out to the last timepoint (week 40) was also evaluated (Figure S1). Similar trends were confirmed using a validated MSD electrochemiluminescence immunoassay (ECLIA) showing increased Trimer 4571-specific antibodies two weeks after regimen completion in all groups (Figure S2).

### Determining antibody specificity following vaccination

**Figure 4:**
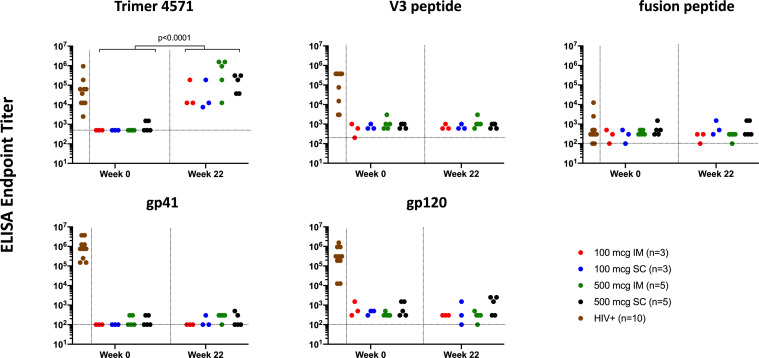
Vaccination with Trimer 4571 induces antibodies against trimer immunogen but not against other Env regions. Anti-Trimer 4571, V3 peptide, fusion peptide (FP), gp120, and gp41 responses at baseline and two weeks after regimen completion were assessed serologically by ELISA. HIV+ human sera were used as assay controls. Horizontal dotted lines indicate the assay negative cut offs. ELISA endpoint titer was defined as the reciprocal of the greatest dilution with an optical density value above 0·1. Significant increase in Trimer 4571 ELISA Endpoint titer was observed from baseline to two weeks after regimen completion across all study groups combined (p<0·0001). IM denotes intramuscular, and SC subcutaneous

We further assessed the specificity of the vaccine-induced antibodies by performing a series of ELISAs against the Trimer 4571 immunogen and specific Env regions including the V3 loop, fusion peptide (FP), gp120, and gp41. As expected, we found a significant increase in Trimer 4571-specific antibodies in all participants two weeks following regimen completion (p<0·0001) (Figure 4). In contrast, no significant increases were observed in antibodies directed against the V3, FP, gp120, or gp41 regions compared to baseline. HIV-positive controls; however, displayed high antibody titers against V3, gp41, and gp120 regions (Figure 4).

### Neutralization activity of vaccine-induced antibodies

We used a panel of 18 Env-pseudoviruses including several BG505 variants and tier 1 and tier 2 pseudoviruses from additional HIV-1 clades to evaluate the neutralization activity of the vaccine-induced antibodies two weeks after regimen completion (Figure S3A). Previous research has shown that the Trimer 4571 immunogen displays low level glycan occupancy at residue 611 near the FP site, and incomplete occupancy at residue 197 near the CD4bs, which may lead to holes in the glycan shield of Trimer 4571.^25^ To determine if this hypoglycosylation affects the induction of neutralizing antibodies, a fully glycosylated autologous BG505 pseudovirus was compared with two BG505 glycosylation mutant pseudoviruses in the panel. BG505.W6M.C2.N611Q contains a mutation which removes a glycosylation site at residue 611, and BG505.W6M.C4.gly4 contains mutations that remove four glycosylation sites around the CD4 binding site. No neutralizing activity was observed against the autologous BG505.W6M.C2.T332N or the majority of heterologous pseudoviruses (Figure S3A). Low levels of neutralizing antibodies were observed in 500 mcg participants against BG505.W6M.C2.N611Q (2 of 5 participants by IM route), BG505.W6M.C4.gly4 (1 of 5 participants by IM route), and the heterologous virus MW965.26 (1 of 5 participants following both IM and SC routes). These results indicate that although the glycan shield limits the development of neutralizing antibodies,^19^ antibodies targeting the hypoglycosylated regions of Trimer 4571 have neutralizing function. Finally, neutralization of CD4-induced epitopes was evaluated using the HIV-2 7312A.V434M virus, which is particularly sensitive to CD4-induced changes.^26^ No neutralization activity was detected in sera against 7312A.V434M in the presence or absence of soluble CD4 two weeks after regimen completion (Figure S3B).

### Antibodies directed against the glycan-free base region of Trimer 4571

**Figure 5:**
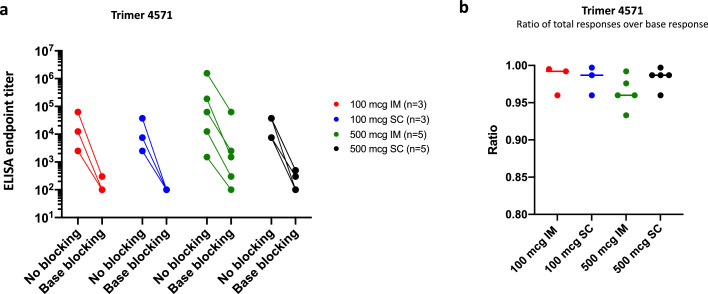
Vaccination with Trimer 4571 elicits antibodies directed against glycan-free trimer base. A) ELISA base-binding endpoint antibody titers in serum were measured with or without trimer base-directed antibodies (RM19R) at two weeks after regimen completion. ELISA endpoint titer was defined as the reciprocal of the greatest dilution with an optical density value above 0·1. B) Ratios of responses directed to base epitopes were calculated as 1 - (endpoint titer with blocking/endpoint titer without blocking), so that the value is 1·00 when all responses are specific to the glycan-free base. IM denotes intramuscular, and SC subcutaneous

Our detection of binding antibodies against the Trimer 4571 immunogen but not the specific Env regions, along with the limited neutralization activity from the participants, led us to examine whether the antibody responses may be focused on the exposed glycan-free base of the soluble Env trimer. This phenomenon was observed in preclinical studies involving stabilized HIV trimer vaccines, including Trimer 4571.^27^^,^^28^ We assessed the proportion of antibody responses directed to the Trimer 4571 base region two weeks after regimen completion with a competition ELISA that added mAb RM19R to block binding to the trimer base region.^27^ Antibodies binding to base epitopes were detected in all participants (Figure 5A) and were the predominant focus of greater than 90% of the antibody response in all participants (Figure 5B). We confirmed these base-directed results using a modified MSD ECLIA (Figure S4A). The base responses to both Trimer 4571-N611Q and -degly4 glycan mutant probes were also evaluated following the observed neutralization activity against the BG505.W6M.C2.N611Q and BG505.W6M.C4.gly4 pseudoviruses. ECLIA analysis revealed high levels of base responses in most participants to both probes (Figure S4B, C). Collectively, this data demonstrates strong base-directed antibody responses following vaccination with Trimer 4571.

### Visualization of antibody binding by EMPEM

**Figure 6:**
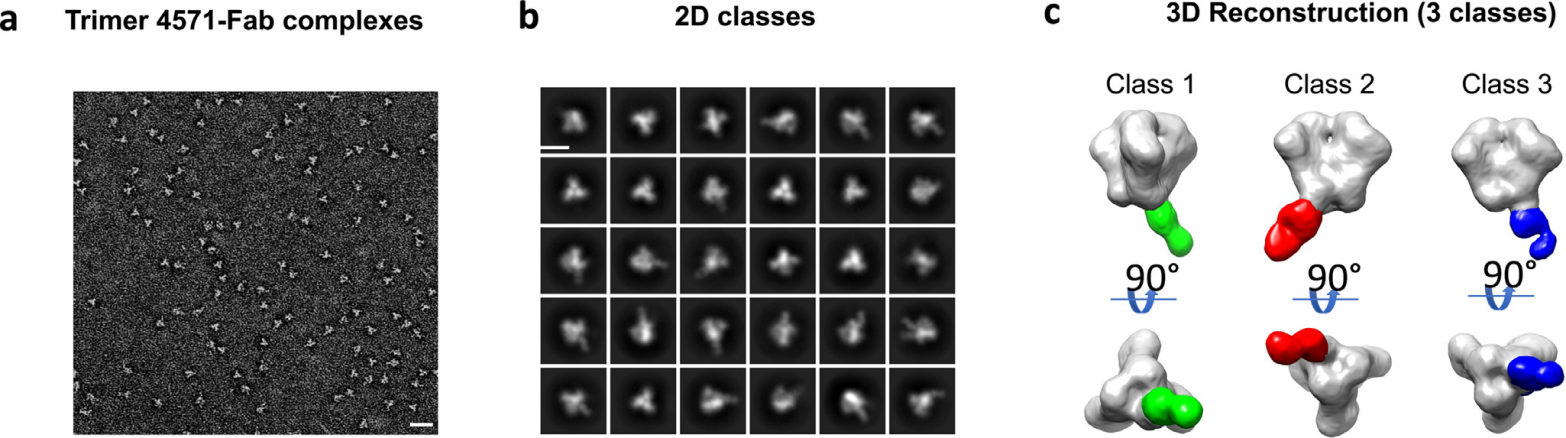
Electron Microscopy Polyclonal Epitope Mapping (EMPEM) of the antibody responses to Trimer 4571 distinguished three different antibody classes. A) Representative micrograph of negatively stained Trimer 4571 and Fab complexes (bar size: 100nm). B) Representative reference-free 2D classes of Fab bound to Trimer 4571 (bar size: 10 nm) C) 3D reconstruction of antibody classes detected at two weeks after regimen completion.

To visualize the specific binding locations and orientations of vaccine-induced antibodies, we performed electron microscopy polyclonal epitope mapping (EMPEM).^27^ For this analysis, we analyzed the serum from the 500 mcg IM dose group participant with the highest levels of Trimer 4571-specific antibodies two weeks after regimen completion. Serum IgG was purified, digested into antigen-binding fragments (Fabs), and bound to Trimer 4571 to form complexes. Antibody-Trimer complexes were purified by size exclusion chromatography and imaged using negative stain electron microscopy (Figures 6A, S5). The 2D class averages revealed complexes with either one or two Fabs bound to Env trimer (Figure 6B). Subsequent 3D reconstructions revealed three dominant classes of complexes, each showing Fab binding at varying angles to the glycan-free base of Trimer 4571 (Figure 6C). This data supports our serologic findings that a significant portion of Trimer 4571-specific antibody responses are directed to the trimer base.

## Discussion

Here we report the results of the first clinical trial in HIV-negative adults evaluating vaccination with an alum adjuvanted HIV-1 Env trimer stabilized in its prefusion-closed conformation. This study demonstrated Trimer 4571 to be safe and well tolerated. Trimer 4571 elicited binding antibodies that were detected by both research and clinical assays, with no indication of CD4-induced conformational change. However, neutralizing activity was limited to the 500 mcg dose groups. Our data suggest that these low neutralization responses may be due to the immunodominance of the glycan-free base and that the highly glycosylated surface of the prefusion-closed trimer has low immunogenicity, supporting the observation that the native prefusion-closed glycosylated Env trimer structure is sheltered from the humoral immune system.^19^

In this trial, vaccination resulted in durable Trimer 4571-specific binding antibodies, with 500 mcg IM administration inducing the highest responses. We measured the antibody responses by both lectin-capture ELISA and MSD ECLIA, the latter utilizing a biotinylated Trimer 4571 immunogen. Since Trimer 4571 was biotinylated at the C-terminus of gp41, this may have led to partial blocking of the detection of base-directed antibodies. Therefore, while the secondary endpoint of the trial focused on the ECLIA assay, we believe that the ELISA assay may be more accurately representing the antibody response following vaccination. Antibody responses against V3 and gp120 epitopes did not increase following vaccination and were significantly lower than those induced by HIV-infection, which is in stark contrast to prior trials of HIV-1 Env protein subunit vaccines.^7^^,^^29^ Our antibody binding analyses also recapitulated preclinical findings showing a high proportion of vaccine-induced antibody responses were directed to the glycan-free base of Trimer 4571.^28^ The glycan-free base region of Trimer 4571 is normally inaccessible to the immune system in the native Env trimer on the viral membrane, and therefore the base-directed antibodies are non-neutralizing. The strong base-directed binding antibody responses may be mitigated in future vaccines by engineering glycans to cover the soluble trimer base to decrease its immunodominance.

While the results of this phase 1 trial aligned with preclinical studies through the production of base-directed antibodies, they differed from preclinical studies by the lack of neutralizing antibodies.^19^^,^^27^^,^^28^ This may be due to inherent immunological differences between humans and the animal models evaluated, and provides further insight into the difficulty in inducing neutralizing antibodies against HIV-1 in humans through vaccination.^30^ Vaccination regimens made up of multiple immunogens may be one strategy to both decrease base responses and increase neutralizing antibodies. This has been achieved in rhesus macaques by the addition of an FP priming immunogen prior to Trimer 4571 boosting.^28^ When vaccination with a stabilized trimer is preceded by vaccination with an FP-carrier conjugate preclinically, the immune response is directed away from the glycan-free base, resulting in FP-directed cross-clade neutralizing antibodies.^28^^,^^31^^,^^32^ Whether these results will be observed in humans remains to be seen, but clinical trials evaluating the impact of FP priming on the Trimer 4751-induced immune response are in development.

Stabilized HIV-1 Env trimers like Trimer 4571 were designed to mimic the glycosylation patterns of naturally occurring HIV-1 BG505 Env.^33^ The glycan shield that assists HIV-1 in evading the immune system therefore remains intact on prefusion-closed conformation trimers resulting in limited exposure of the neutralizing epitopes on the vaccine immunogen.^19^ This may in part explain the low neutralization activity following Trimer 4571 vaccination. This high degree of glycan shielding is in contrast to other class 1 fusion proteins, such as respiratory syncytial virus, where stabilized pre-fusion protein-based vaccines have resulted in high levels of neutralizing antibodies.^16^ Interestingly, the neutralization responses observed in this trial occur predominantly against pseudoviruses with mutations that create holes in the Env glycan shield, which further supports the hypothesis that glycosylation plays a role in the low neutralizing activity observed.

Another factor known to influence the immunogenicity of Trimer 4571 in preclinical studies is the choice of adjuvant. Rhesus macaques immunized with Trimer 4571 adjuvanted with Adjuplex (a carbomer-lecithin homopolymer) showed greater neutralizing responses compared to animals receiving Trimer 4571 adjuvanted with alum.^19^ Our clinical trial, for which safety and tolerability were primary outcomes, utilized alum as an adjuvant due to its well-established safety profile in numerous licensed vaccines.^34^ The reassuring safety data from this trial supports stabilized trimers being evaluated with higher potency adjuvants to further enhance immunogenicity in planned future trials.

There are limitations to this phase 1 trial that are important to consider when planning future trials. The small number of participants limits the power of the statements we can make from our analyses, but this trial nevertheless provides valuable human data for the iterative design process toward developing an effective HIV-1 vaccine. The positive VISP results observed in several participants in this trial were monitored closely and social impacts were avoided through counselling. While VISP was not an unexpected occurrence, it does highlight the need to mitigate any social risks from inaccurate HIV diagnoses that trial participants may experience during future trial experiences and beyond.

This trial demonstrates that a prefusion-closed stabilized HIV-1 Env trimer adjuvanted with alum is safe and immunogenic. Our results also show that the stabilization of Trimer 4571 blocked CD4-induced responses observed with previous gp120 protein vaccines and represents an advancement in the search for a stabilized HIV-1 Env protein vaccine immunogen. However, further steps are needed to improve Trimer 4571-induced neutralization overall. This trial is the first step in an approach to improve immunogenicity outcomes for both immunogen and vaccination regimen design strategies. This trial demonstrated the safety of the stabilized timer immunogen and provided us with knowledge on the individual impact of Trimer 4571 on the immune response. The next steps in this approach will involve the use of epitope-focused immunogens such as HIV-1 FP as a prime followed by a boost with stabilized Env trimers and utilization of high potency adjuvants.^22^ Trimer 4571 is also being evaluated in alternative approaches as well. Currently, a prime-boost strategy being evaluated employs an Ad4-HIV-1 Env prime followed by Trimer 4571 boost (NCT03878121) in healthy, HIV-1-negative adults. Another trial (NCT04985760) will evaluate Trimer 4571 in HIV-1 infected adults on antiretroviral therapy. Furthermore, other stabilized HIV-1 trimers with distinct stabilizing mutations are also being evaluated (NCT03699241). Collectively the data from this trial and others serve as contributing steps in the effort to develop an effective HIV-1 vaccine.

## Contributors

MRG is the principal investigator of VRC 018. MRG, RSR, NMB, SH, MC-C, SV, OT, SP, MN, AC, CB, KC, JG, BSG, RAK, ABM, JRM, PDK, JEL contributed to conception and design. MRG, LN, CSH, IJG, CTC, ATW contributed to investigation and sample collection. KVH, MRG, MH, SN, RV, RW, EEC, LS, JS, CLC, MN, AB, AC, CC, ARC, HD, HG, DKP, ES, I-TT, TZ, SO, BF, NAD-R, ABM, PDK contributed to data analysis and interpretation. All authors contributed to manuscript writing and final approval of the manuscript. KVH, MRG, and MH all have accessed and verified the underlying data for this trial.

## Data sharing statement

Data generated in this study is available as de-identified data on ClinicalTrials.gov, NCT03783130. The study protocol, statistical analysis plan, and informed consent form are available on ClinicalTrials.gov (https://clinicaltrials.gov/ProvidedDocs/30/NCT03783130/Prot_SAP_ICF_000.pdf). The raw immunological data is available in Supplementary material 2 at time of publication. Additional data may be made available upon request to the corresponding author.

## Declaration of interests

CC, HG, JRM, and PDK are listed on patent applications involving Trimer 4571. All other authors declare no competing interests.

## Appendix Supplementary materials

Image, application 1

Image, application 2

Image, application 3
