# Supplementary material for: Safety and immunogenicity of an HIV-1 prefusion-stabilized envelope trimer (Trimer 4571) vaccine in healthy adults: A first-in-human open-label, randomized, dose-escalation, phase 1 clinical trial
Source: eClinicalMedicine. 2022 Jun 1;48:101477. doi: 10.1016/j.eclinm.2022.101477 (PMC9249552; doi:10.1016/j.eclinm.2022.101477)
Supplement: Supplementary file 1 [file mmc1.docx]

**Supplementary material 1:** Supplemental methods, tables, figures, and trial protocol

**Supplementary material 2:** Raw subject level results for each immunological assay included within the publication
