# Supplementary material for: Safety and immunogenicity of an HIV-1 prefusion-stabilized envelope trimer (Trimer 4571) vaccine in healthy adults: A first-in-human open-label, randomized, dose-escalation, phase 1 clinical trial
Source: eClinicalMedicine. 2022 Jun 1;48:101477. doi: 10.1016/j.eclinm.2022.101477 (PMC9249552; doi:10.1016/j.eclinm.2022.101477)
Supplement: Supplementary file 2 [file mmc2.pdf]

Supplemental methods, tables, and figures for “Safety and immunogenicity of an HIV-1 prefusion-stabilized envelope trimer (Trimer 4571) vaccine in healthy adults: A randomized phase 1 clinical trial” by Houser and Gaudinski et al

Table of Contents

VRC 018 Study Team members	3
Supplemental Methods	4-10
Figure S1	11
Figure S2	12
Figure S3	13
Figure S4	14
Figure S5	15
Table S1	16
Table S2	17
Table S3	18
References	19-20

VRC 018 Study Team members not listed as authors include: Grace Chen, Cristina Carter, Joseph Casazza, LaSonji Holman, Abidemi Ola, Karen Parker, Allison Beck, Anita Arthur, Jamie Saunders, Pamela Costner, Jennifer Cunningham, Aba Eshun, Brenda Larkin, Floreliz Mendoza, Xiaolin Wang, William Whalen, Maria Claudia Burgos Florez, Galina Yamshchikov, Olga Vasilenko, Iris Pittman, Eugenia Burch, Lam Le, Carmencita Graves, Catina Evans, Renunda Hicks, Preeti Apte, Pernell Williams, La'Shawn Requilman, Thuy Nguyen, Colin Tran, Morgan Anderson, Justine Jones, Shinyi Telscher, Zana Blaku, Jihyun Esther Jeon, Keva Dillard, Srilatha Edupuganti, Flavio Matassoli, Mike Castro, Chris Gonelli, Christian Hatcher, Obrimpong Amoa-Awua, Rosa Silva, Jagada Thillainathan, Shiuishu Wang, Mark Louder, Sijy O'Dell, Stephen Schmidt, Krisha McKee, Yaroslav Tsybovsky. **The VRC Vaccine Production Program (VPP) members**: Nadia Amharref, Gabriel Arias, Nathan Barefoot, Michael F. Bender, Elizabeth Carey, Alegria T. Caringal, Juliane Carvalho, Naga Chalamalasetty, Adam Charlton, Rajoshi Chaudhuri, Mingzhong Chen, Peifeng Chen, Nicole Cibelli, Jonathan W. Cooper, Hussain Dahodwala, Marianna Fleischman, Haley Fuller, Julia Frederick, Isaac Godfrey, Deepika Gollapudi, Daniel Gowetski, Krishana Gulla, Joe Horwitz, Althaf Hussain, Vera Ivleva, Tina Khin , Lisa Kueltzo, Nadji Lambert, Q. Paula Lei, Yile Li, Aakash Patel, Daniel Schankel, Liz Scheideman, Nicole A. Schneck, Zachary Schneiderman, Andrew Shaddeau, William Shadrick, Renata Skubutyte, Brad Tippett , Venkata Mangalampalli, Gabriel Moxey, Karen Vickery, Sara Witter Zafar, Yaqui (Tina) Zhang, Erwin Rosales-Zavala, Alison Vinitsky, Xin Wang, Jack G. Yang, Yanhong Yang, Thomas Smith III, Janel Holland-Linn.

Supplemental Methods:

HIV-positive serum samples

HIV-positive sera collected under the IRB-approved clinical trial protocols VRC 200 (ClinicalTrials.gov NCT00067054) and VRC PAN (ClinicalTrials.gov NCT00027482) were used as positive controls for immunological assays.

Production of HIV-1 Probes

The antigen probes used for the analyses were produced as previously described with minor modifications.[1,2] Probes were made for Trimer 4571 (BG505 DS-SOSIP), Trimer 4571 with a mutation to remove a glycosylation site at residue 611 near the fusion peptide (N611Q), Trimer 4571 with mutations to remove four glycosylation sites surrounding the CD4bs at residues 197, 276, 462, and 362 (degly4), and monomeric MW965 gp120. Briefly, C-terminal AVI-tagged Trimer 4571 (BG505 DS-SOSIP FPv1 10lnQQ-AVI), Trimer 4571 mutants (BG505 DS-SOSIP N611Q 10lnQQ-AVI and BG505 DS-SOSIP degly4 10lnQQ-AVI), or monomeric MW965 gp120 (MW965 gp120 10lnQQ-AVI) were produced using transient transfection in FreeStyle 293F cells (ThermoFisher Scientific) by pre-mixing the DNA with Turbo293 transfection reagent (Speed BioSystems). Constructs encoding trimers were co-transfected with furin plasmid DNA. The cells were cultured for 6 days before AVI-tagged BG505 DS-SOSIP trimer was purified from supernatant using VRC01 affinity chromatography, followed by a Superdex 200 16/600 gel filtration column (Cytiva) and negatively selected using a 447-52D affinity column to remove V3-exposed trimers (developed by VRC), as described previously.[1] BG505 DS-SOSIP FPv1

10lnQQ-AVI protein was biotinylated with BirA enzyme (Avidity) overnight and purified by gel filtration chromatography.

AVI-tagged MW965 gp120 protein and gp140 mutants (BG505 DS-SOSIP N611Q and BG505 DS-SOSIP degly4) also included an N-terminal single-chain Fc (scFc) tag, in tandem with an HRV3C cleavage site. For scFc-tagged proteins, supernatant was incubated with Protein A resin (Cytiva) before the resin was collected and washed. The captured AVI-tagged proteins were then concurrently biotinylated with BirA enzyme and cleaved from the Fc purification tag with HRV3C protease (Cytiva). After overnight incubation, the liberated and biotinylated protein was collected, concentrated using Amicon Ultra-15-30K (Millipore), and applied to a Superdex 200 16/600 gel filtration column equilibrated with PBS. For the gp120 probe, the fractions were pooled and concentrated directly after gel filtration. For the gp140 variant probes, the corresponding peak fractions were pooled and subjected to negative selection with a V3 cocktail column (developed by VRC) containing six V3-directed antibodies (1006-15D, 2219, 2557, 2558, 3074, and 50·1) to remove V3-exposed trimers,[3-7] followed by concentration. The probes were stored in PBS containing 10% glycerol in -80˚C.

Trimer 4571 Enzyme-linked immunosorbent assay (ELISA)

Anti-Trimer 4571 antibodies were evaluated by ELISA using lectin-captured HIV-1 Trimer 4571, as previously described.[8] Briefly, 96-well half area plates (Costar High Binding Half-Area; Corning, Kennebunk, ME) were coated overnight with snowdrop lectin from *Galanthus nivalis* (Sigma-Aldrich, St. Louis, MO), blocked, and incubated, followed by addition of Trimer 4571. Next, 5-fold serially diluted sera, starting at 1:100, was added to the plates. Following

incubation, goat anti-human IgG conjugated to horseradish peroxidase (Jackson Immunoresearch, West Grove, PA) diluted 1:5000 was added. Plates were washed, developed with tetramethylbenzidine (TMB) substrate (SureBlue; KPL, Gaithersburg, MD) and read at 450 nm (SpectraMax using SoftMax Pro, V5; Molecular Devices, Sunnyvale, CA). Optical densities (OD) were analyzed following background subtraction. The endpoint titer was defined as the reciprocal of the greatest dilution with an OD value above 0·1.

For evaluation of base-binding antibody responses, one additional step was added to the assay, as previously described.[9] Prior to adding serially diluted samples, plates were washed and then incubated with either 2 µg/ml RM19R Fab/blocking buffer that blocked binding to Trimer 4571 base region but did not interfere with other conserved regions (base blocking) or blocking buffer alone (no base blocking).

ELISAs for fusion peptide (FP)-, V3 peptide-, gp120-, and gp41-specific antibodies

ELISAs were used to detect epitope-specific antibodies as previously described.[8] Briefly, streptavidin coated plates were washed and incubated with one of the following: fusion peptide FP8-PEG12-biotin (AVGIGAVF-PEG12, GenScript, Piscataway, NJ), V3 peptide with the sequence of TRPNNNTRKSIRIGPGQAFYATGDIIGDIRQAH (GenScript, Piscataway, NJ), gp120 protein derived from MW965, or gp41 protein from BH10 obtained from Abcam (Cat# ab49070, Cambridge, UK). Sera, heat-inactivated at 56°C for one hour, were assessed at five-fold dilutions starting at 1:200 for V3 and 1:100 for FP, gp120, and gp41 epitopes. Plates were incubated, washed, treated with goat anti-human IgG HRP (Jackson Immunoresearch, West Grove, PA) and developed with TMB substrate (SureBlue, KPL, Gaithersburg, MD). The reaction

was stopped with 1N sulfuric acid (Fisher Scientific Fairlawn, NJ cat#201670). Plates were then read at 450 nm on a microplate spectrophotometer (SpectraMax using SoftMax Pro, V5; Molecular Devices, Sunnyvale, CA). The endpoint titer for each sample was defined as the reciprocal of the greatest dilution with an OD value above 0·1. FP-directed antibody VRC34.01 was used as the positive control for the anti-FP ELISA[10].

Trimer 4571 Electrochemiluminescence immunoassay (ECLIA)

Serum samples were analyzed for the presence of Trimer 4571-specific antibodies by a qualified ECLIA with the Meso Scale Discovery (MSD) platform.[9] Briefly, duplicate 8-fold serially diluted samples were added to standard MSD 384 well plates coated with biotinylated BG505 DS-SOSIP.664 FPv1 10lnQQ-AVI protein. Following incubation and a wash, secondary SULFO-TAG conjugated goat anti-human antibody (Meso Scale Discovery) was used to detect Trimer 4571-specific antibodies. Plates were incubated, washed, and read using the MSD SECTOR® Imager 6000 (Meso Scale Discovery). Area under the curve (AUC) was calculated from the raw electrochemiluminescence (ECL) sample response over eight-fold dilution series (dilution factor) for each sample. Serum samples from HIV-positive individuals were used as positive controls, and the assay negative cut off was determined by screening naïve human sera samples. A similar, non-qualified analysis was conducted using biotinylated AVI-tagged Trimer 4571-N611Q and Trimer 4571-degly4 probes.

Base-Binding Trimer 4571 Antibody ECLIA

We utilized the MSD platform to perform a modified ECLIA assay specifically designed to capture trimer base-binding responses as previously described.[9] Briefly, duplicate four-fold serially diluted samples were added to standard MSD streptavidin plates coated individually with biotinylated AVI-tagged Trimer 4571, Trimer 4571-N611Q, or Trimer 4571-degly4. Samples were then incubated with MSD Blocker A (Meso Scale Discovery) either alone or with trimer base-directed mouse mAb cocktail (1:1 ratio of RM20A2 and RM19R with mouse Fc) (developed by VRC).[11] Secondary SULFO-TAG conjugated goat anti-mouse antibody (Meso Scale Discovery) or SULFO-TAG conjugated goat anti-Hu/NHP antibody (Meso Scale Discovery) was used for detection. Plates were then washed and read using 1X MSD Read Buffer on the MSD SECTOR® Imager (Meso Scale Discovery).

Neutralization Assay

HIV-1 virus neutralization assay was performed by utilizing a single-round of HIV-1 Env pseudovirus infection of TZM-bl target cells as previously described[12]. Briefly, HIV-1 Env pseudoviruses were generated by transfecting 293T/17 cells with optimized ratios of envelope-expressing plasmid and backbone vector (pSG3ΔEnv). A panel of 18 tier 1 and tier 2 pseudoviruses was tested for neutralizing activity, including clade A (BG50.W6M.C2.T332N, autologous virus), clade A (BG505.W6M.C2.N611Q, highly susceptible to fusion peptide-directed Abs), clade A (BG505.W6M.C2.gly4, highly susceptible to CD4bs-directed Abs), clade A (398-F1.F6.20.SG3, Q23.17), clade AC (246-F3.C10.2.SG3), clade AE (CNE55.SG3, CNE8.SG3), clade AG (T250-4), clade B (TRO.11.SG3, X2278.C2.B6.SG3), clade BC (BJOX002000.03.2., CH119.10.SG3), clade C (25710-2.43.SG3, Ce1176.A3.SG3, Ce703010217.B6.SG3, MW965.26),

clade G (X1632.S2.B10.SG3), and non-HIV (SIVmac251.30.SG3). Neutralization by CD4-induced antibodies was assessed through the HIV-2 strain 7312A.434M, with or without pre-treatment for with 0·5ug/ml soluble CD4 (sCD4) for 1 hour, as previously described.[13] Since the 7321 strain is a live virus, indinavir is added to restrict the virus to a single round of replication. Positive and negative controls were included in all assays. Experimental results were displayed as serum dilutions that produced 50% neutralization (ID_{50}) against the panel of viruses tested.

IgG purification, Fab preparation, and Electron Microscopy Polyclonal Epitope Mapping (EMPEM)

To obtain polyclonal IgGs, 1ml serum samples were heat inactivated at 55°C for 30 minutes, incubated overnight with packed Protein G resin (Cytiva) using a 1:1 ratio of serum to resin, and washed. Bound IgGs were eluted (Pierce IgG elution buffer) and immediately neutralized using 10% 1M Tris, pH 8·5. Purified IgGs were cleaved using a Fab preparation kit with immobilized papain (ThermoFisher #44985) following manufacturer protocol. To remove Fc fraction, the cleaved mixture was incubated with Protein A (Cytiva). The flowthrough, containing Fabs, was then added to a Superdex 200 Increase column (Cytiva) and Fab fractions were pooled and concentrated to 1mg/ml, then stored at 4°C. To prepare complexes for EM, 1 ml polyclonal Fabs were incubated with 10 µg Trimer 4571 overnight at room temperature and then added to a Superdex 200 Increase column. The peak corresponding to Fab-Trimer 4571 complexes were pooled and concentrated to 50 ug/ml. To prepare the grids, Fab-Trimer 4571 complexes were diluted to 10ug/ml and applied to glow discharged carbon-coated Cu grids (200-mesh, Electron Microscopy Sciences #CF200-CU), blotted with filter paper, and washed. Negative stain (uranyl-

formate 2%, Electron Microscopy Sciences #22451) was applied to the grids three times with blotting. Data were collected on a Talos F200C electron microscope (ThermoScientific) operating at 200 keV with magnification set at 57,000x, corresponding to a pixel size of 2·53 A. Fifty micrographs were collected at four tilt angles (0, 15, 30 and 45 degrees). Data were processed using CryoSPARC software platform.[14] Approximately 60,000 particles were picked and 2D classified. Initial models were generated using Ab inito routine in cryoSPARC. Each class with at least one Fab bound was further refined using Homogenous Refinement routine. Maps were visualized and segmented in UCSF Chimera.[15]

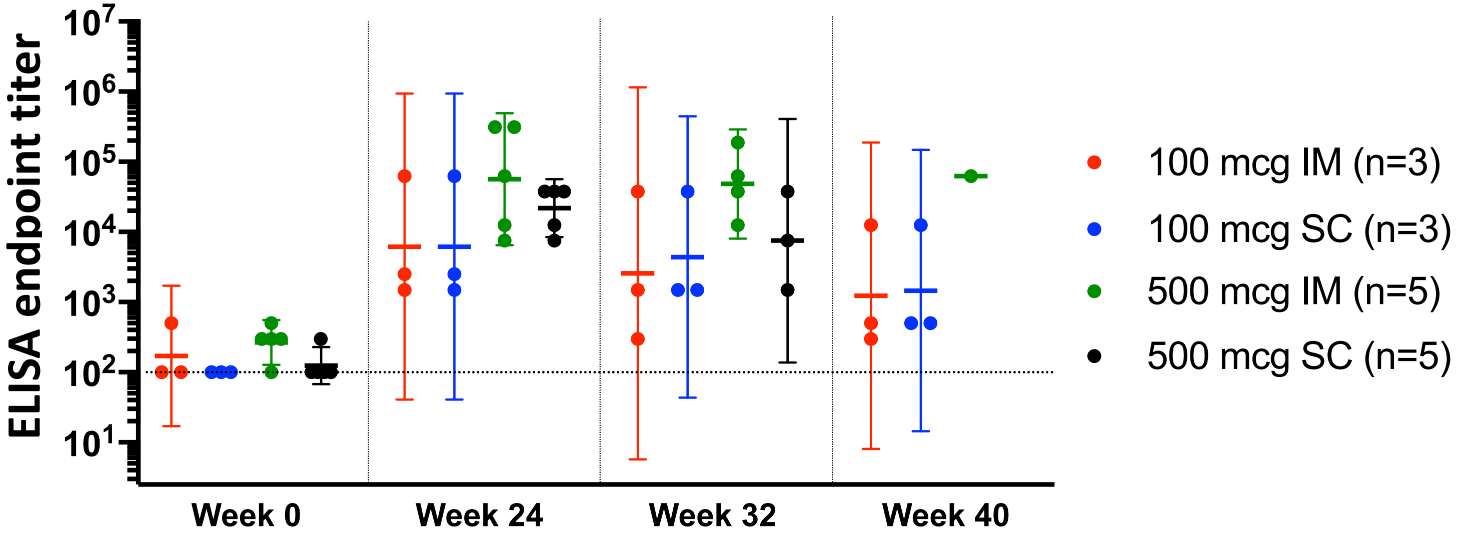

Figure S1. Vaccination with Trimer 4571 induces durable trimer-specific antibody responses by ELISA. Trimer 4571-specific antibody titers in serum were measured by ELISA at baseline at weeks 24, 32, and 40 following each vaccination regimen. Geometric mean titers (GMTs) with 95% confidence intervals (CIs) are indicated by error bars. Horizontal dotted line indicates the assay negative cut off. ELISA endpoint titer was defined as the reciprocal of the greatest dilution with an optical density value above 0.1. Due to COVID-19 pandemic sample collection was impacted at weeks 32 and 40 (detailed in Table S2). IM denotes intramuscular, and SC subcutaneous.

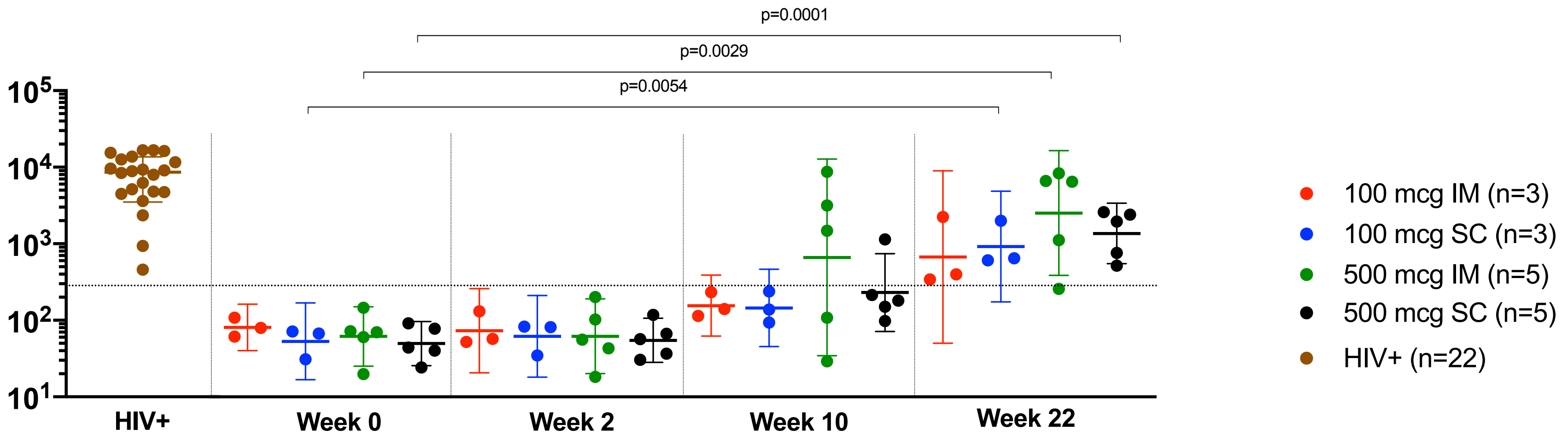

Figure S2. Vaccination with Trimer 4571 induces trimer-specific antibody responses by ECLIA. Trimer 4571 specific antibody titers were measured by a validated MSD electrochemiluminescence immunoassay (ECLIA). Area Under the Curve (AUC) was calculated from the raw electrochemiluminescence (ECL) sample response over 8-fold dilution series (dilution factor) for each sample. HIV+ human sera were used as positive controls. Horizontal dotted line indicates the assay negative cut off determined by screening naïve human sera. Arrows indicate vaccination time points. Geometric mean titers (GMTs) with 95% confidence intervals (CIs) are indicated by error bars. Significant differences from baseline to two weeks after regimen completion were observed for 100 mcg SC (p=0·0054), 500 mcg IM (p=0·0029), and 500 mcg SC (p=0·0001) groups. IM denotes intramuscular, and SC subcutaneous.

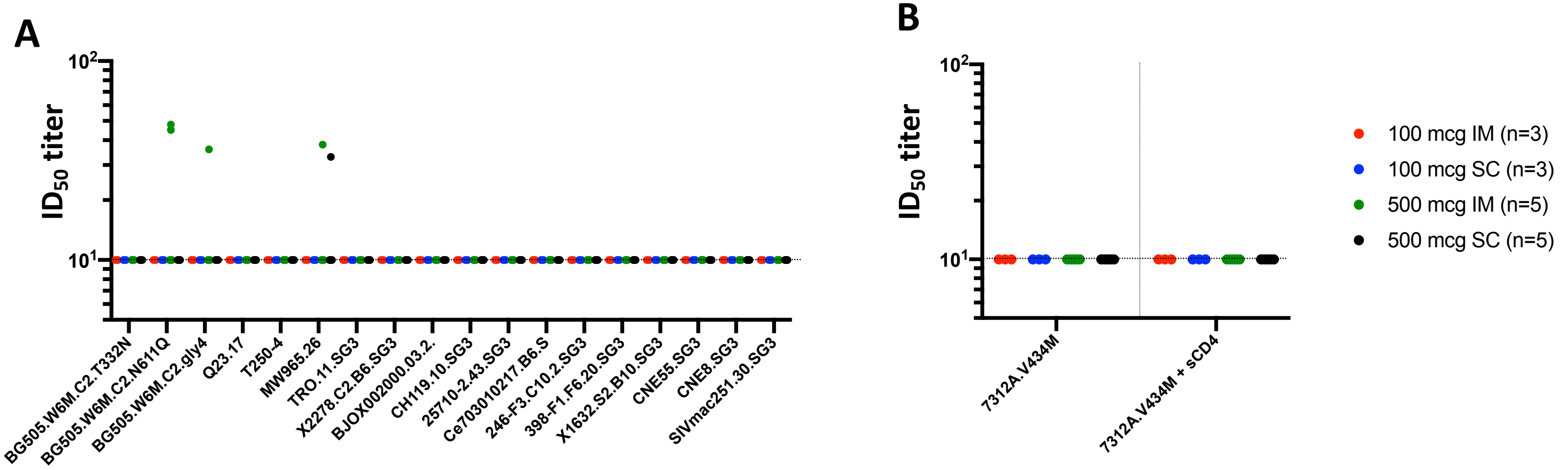

Figure S3. Limited neutralizing antibodies are observed, with no indication of neutralization against CD4-induced epitopes after vaccination with Trimer 4571. A) Serum neutralization (ID_{50}) titers against 18 HIV-1 Env pseudoviruses were evaluated at two weeks after regimen completion. B) Serum neutralization (ID_{50}) titers by CD4-induced antibodies were assessed by HIV-2 7312A.V43M virus, in the presence and absence of sCD4, at two weeks after regimen completion. Limit of detection for the assay is indicated by the dotted line. IM denotes intramuscular, and SC subcutaneous.

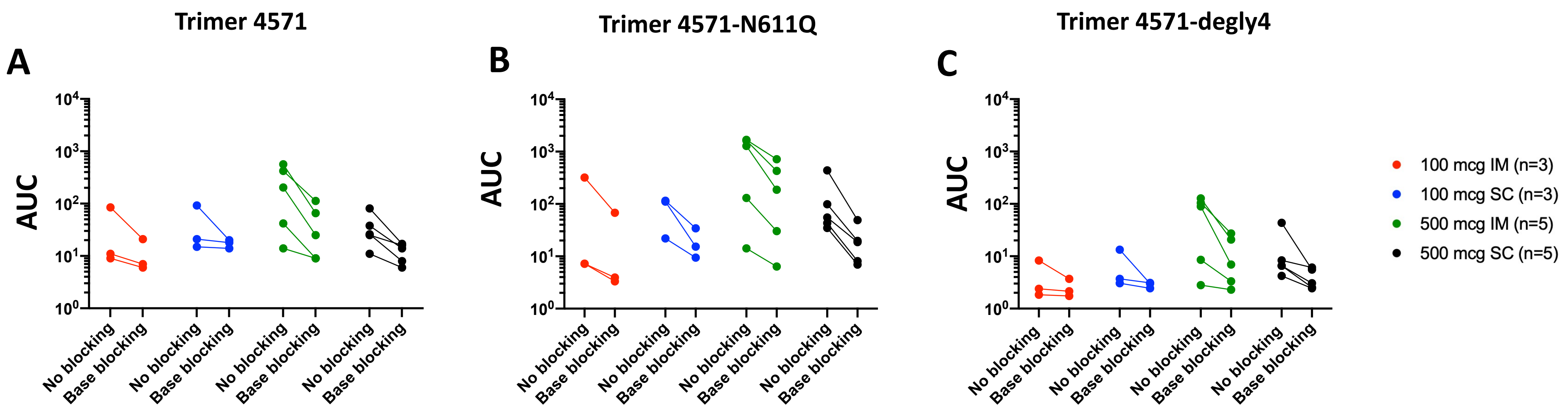

Figure S4. Vaccination with Trimer 4571 results in responses directed against glycan-free trimer base. A) Trimer 4571 specific base-binding antibodies. B) Base-directed antibodies against Trimer 4571-N611Q glycan mutant. C) Base-directed antibodies against Trimer 4571-degly4 glycan mutant. Trimer 4571, Trimer 4571- N611Q, and Trimer4571-degly4 antibodies in serum were detected by a modified ECLIA with or without trimer base-directed antibodies at two weeks after regimen completion. Area Under the Curve (AUC) was calculated from the raw electrochemiluminescence (ECL) sample response over 8-fold dilution series (dilution factor) for each sample. IM denotes intramuscular, and SC subcutaneous.

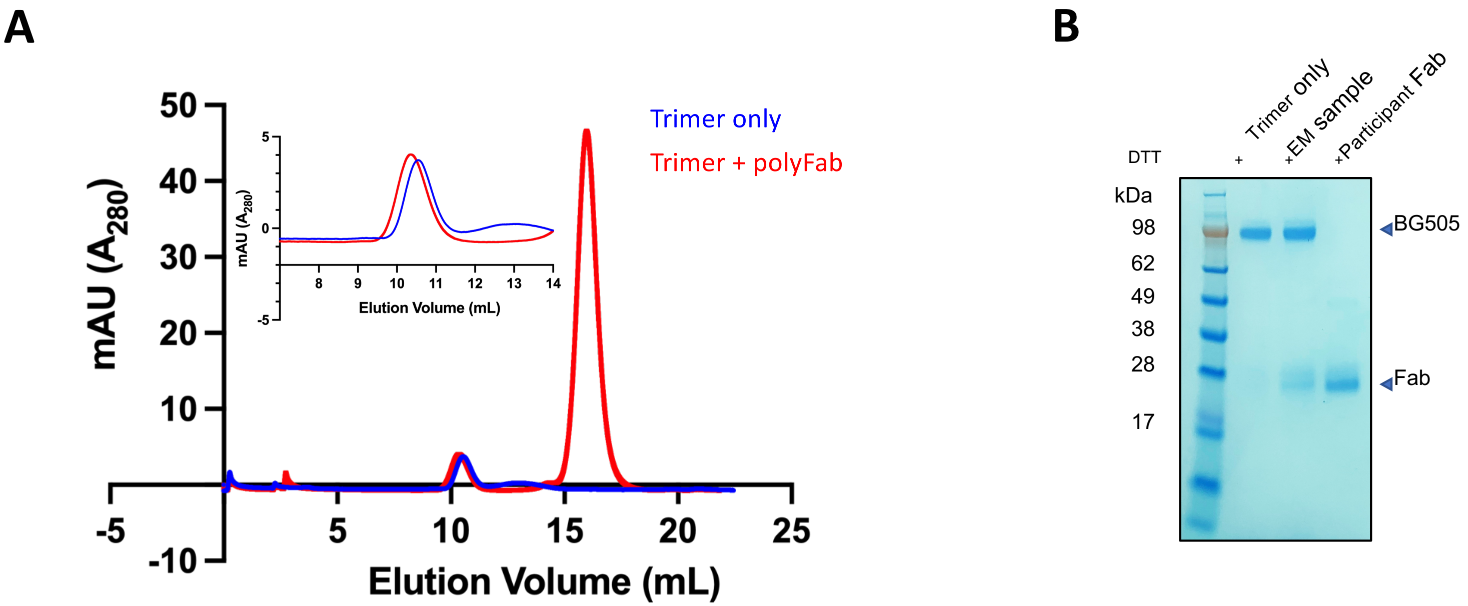

Figure S5. Purification of Trimer 4571 in complex with polyclonal Fab. A) Representative antibody purification profile where IgG is extracted form serum sample and digested with papain to Fab and SDS-Page for Trimer 4571 and Fab complexes from a 500 mcg IM dose participant. B) SDS-PAGE (reducing condition) of the peaks in chromatogram. Peaks at 11mL (EM sample) were concentrated to a similar level as peak at 16mL (participant Fab) before loading.

Table S1. COVID-19 Study visits impact. In-person clinic follow up visits interrupted due to COVID-19 pandemic were conducted by phone to assess safety signals in study participants from the 500 mcg dose groups, and no samples were collected.

Subject	Group	Product Administration Discontinued	Visits Where Phone Calls Were Conducted to Assess Safety Signals
8	3	No	Week 40
9	3	No	Week 40
10	3	No	Week 40
11	3	No	Week 32, 40
13	4	No	Week 40
14	4	No	Week 40
15	4	No	Week 32, 40
16	4	No	Week 32, 40

Table S2. Maximum local and systemic reactogenicity reported within 7 days after receipt of each Trimer 4571 vaccination according to dose and route of administration.

	100 mcg IM (N=3)			100 mcg SC (N=3)			500 mcg IM (N=5)			500 mcg SC (N=5)		
**Symptoms**	**Vaccination 1**	**Vaccination 2**	**Vaccination 3**	**Vaccination 1**	**Vaccination 2**	**Vaccination 3**	**Vaccination 1**	**Vaccination 2**	**Vaccination 3**	**Vaccination 1**	**Vaccination 2**	**Vaccination 3**
**PAIN/TENDERNESS**												
None	1 (33·3%)	2 (66·7%)	2 (66·7%)	1 (33·3%)	1 (33·3%)	1 (33·3%)	0 (0%)	0 (0%)	1 (20·0%)	2 (40·0%)	1 (20·0%)	2 (40·0%)
Mild	2 (66·7%)	1 (33·3%)	1 (33·3%)	2 (66·7%)	2 (66·7%)	2 (66·7%)	5 (100%)	5 (100%)	4 (80·0%)	3 (60·0%)	4 (80·0%)	3 (60·0%)
Moderate	0 (0%)	0 (0%)	0 (0%)	0 (0%)	0 (0%)	0 (0%)	0 (0%)	0 (0%)	0 (0%)	0 (0%)	0 (0%)	0 (0%)
Severe	0 (0%)	0 (0%)	0 (0%)	0 (0%)	0 (0%)	0 (0%)	0 (0%)	0 (0%)	0 (0%)	0 (0%)	0 (0%)	0 (0%)
**REDNESS**												
None	3 (100%)	3 (100%)	3 (100%)	3 (100%)	2 (66·7%)	1 (33·3%)	5 (100%)	5 (100%)	4 (80·0%)	4 (80·0%)	4 (80·0%)	4 (80·0%)
Mild	0 (0%)	0 (0%)	0 (0%)	0 (0%)	1 (33·3%)	0 (0%)	0 (0%)	0 (0%)	0 (0%)	1 (20·0%)	1 (20·0%)	0 (0%)
Moderate	0 (0%)	0 (0%)	0 (0%)	0 (0%)	0 (0%)	2 (66·7%)	0 (0%)	0 (0%)	1 (20·0%)	0 (0%)	0 (0%)	0 (0%)
Severe	0 (0%)	0 (0%)	0 (0%)	0 (0%)	0 (0%)	0 (0%)	0 (0%)	0 (0%)	0 (0%)	0 (0%)	0 (0%)	1 (20·0%)
**SWELLING**												
None	3 (100%)	3 (100%)	3 (100%)	3 (100%)	2 (66·7%)	1 (33·3%)	5 (100%)	4 (80·0%)	4 (80·0%)	5 (100%)	5 (100%)	4 (80·0%)
Mild	0 (0%)	0 (0%)	0 (0%)	0 (0%)	1 (33·3%)	1 (33·3%)	0 (0%)	0 (0%)	0 (0%)	0 (0%)	0 (0%)	0 (0%)
Moderate	0 (0%)	0 (0%)	0 (0%)	0 (0%)	0 (0%)	1 (33·3%)	0 (0%)	1 (20·0%)	1 (20·0%)	0 (0%)	0 (0%)	1 (20·0%)
Severe	0 (0%)	0 (0%)	0 (0%)	0 (0%)	0 (0%)	0 (0%)	0 (0%)	0 (0%)	0 (0%)	0 (0%)	0 (0%)	0 (0%)
**ANY LOCAL SYMPTOM**												
None	1 (33·3%)	2 (66·7%)	2 (66·7%)	1 (33·3%)	1 (33·3%)	1 (33·3%)	0 (0%)	0 (0%)	1 (20·0%)	2 (40·0%)	1 (20·0%)	2 (40·0%)
Mild	2 (66·7%)	1 (33·3%)	1 (33·3%)	2 (66·7%)	2 (66·7%)	0 (0%)	5 (100%)	4 (80·0%)	3 (60·0%)	3 (60·0%)	4 (80·0%)	2 (40·0%)
Moderate	0 (0%)	0 (0%)	0 (0%)	0 (0%)	0 (0%)	2 (66·7%)	0 (0%)	1 (20·0%)	1 (20·0%)	0 (0%)	0 (0%)	0 (0%)
Severe	0 (0%)	0 (0%)	0 (0%)	0 (0%)	0 (0%)	0 (0%)	0 (0%)	0 (0%)	0 (0%)	0 (0%)	0 (0%)	1 (20·0%)
**HEADACHE**												
None	3 (100%)	3 (100%)	2 (66·7%)	2 (66·7%)	3 (100%)	2 (66·7%)	4 (80·0%)	4 (80·0%)	4 (80·0%)	4 (80·0%)	5 (100%)	5 (100%)
Mild	0 (0%)	0 (0%)	1 (33·3%)	1 (33·3%)	0 (0%)	1 (33·3%)	1 (20·0%)	1 (20·0%)	1 (20·0%)	1 (20·0%)	0 (0%)	0 (0%)
Moderate	0 (0%)	0 (0%)	0 (0%)	0 (0%)	0 (0%)	0 (0%)	0 (0%)	0 (0%)	0 (0%)	0 (0%)	0 (0%)	0 (0%)
Severe	0 (0%)	0 (0%)	0 (0%)	0 (0%)	0 (0%)	0 (0%)	0 (0%)	0 (0%)	0 (0%)	0 (0%)	0 (0%)	0 (0%)
**MALAISE**												
None	3 (100%)	3 (100%)	3 (100%)	2 (66·7%)	2 (66·7%)	2 (66·7%)	3 (60·0%)	4 (80·0%)	5 (100%)	3 (60·0%)	5 (100%)	5 (100%)
Mild	0 (0%)	0 (0%)	0 (0%)	0 (0%)	1 (33·3%)	1 (33·3%)	2 (40·0%)	1 (20·0%)	0 (0%)	2 (40·0%)	0 (0%)	0 (0%)
Moderate	0 (0%)	0 (0%)	0 (0%)	1 (33·3%)	0 (0%)	0 (0%)	0 (0%)	0 (0%)	0 (0%)	0 (0%)	0 (0%)	0 (0%)
Severe	0 (0%)	0 (0%)	0 (0%)	0 (0%)	0 (0%)	0 (0%)	0 (0%)	0 (0%)	0 (0%)	0 (0%)	0 (0%)	0 (0%)
**MYALGIA**												
None	2 (66·7%)	2 (66·7%)	2 (66·7%)	3 (100%)	3 (100%)	3 (100%)	3 (60·0%)	3 (60·0%)	5 (100%)	5 (100%)	4 (80·0%)	5 (100%)
Mild	1 (33·3%)	1 (33·3%)	1 (33·3%)	0 (0%)	0 (0%)	0 (0%)	2 (40·0%)	2 (40·0%)	0 (0%)	0 (0%)	1 (20·0%)	0 (0%)
Moderate	0 (0%)	0 (0%)	0 (0%)	0 (0%)	0 (0%)	0 (0%)	0 (0%)	0 (0%)	0 (0%)	0 (0%)	0 (0%)	0 (0%)
Severe	0 (0%)	0 (0%)	0 (0%)	0 (0%)	0 (0%)	0 (0%)	0 (0%)	0 (0%)	0 (0%)	0 (0%)	0 (0%)	0 (0%)
**NAUSEA**												
None	3 (100%)	3 (100%)	3 (100%)	2 (66·7%)	3 (100%)	3 (100%)	5 (100%)	5 (100%)	5 (100%)	4 (80·0%)	5 (100%)	5 (100%)
Mild	0 (0%)	0 (0%)	0 (0%)	0 (0%)	0 (0%)	0 (0%)	0 (0%)	0 (0%)	0 (0%)	1 (20·0%)	0 (0%)	0 (0%)
Moderate	0 (0%)	0 (0%)	0 (0%)	1 (33·3%)	0 (0%)	0 (0%)	0 (0%)	0 (0%)	0 (0%)	0 (0%)	0 (0%)	0 (0%)
Severe	0 (0%)	0 (0%)	0 (0%)	0 (0%)	0 (0%)	0 (0%)	0 (0%)	0 (0%)	0 (0%)	0 (0%)	0 (0%)	0 (0%)
**ANY SYSTEMIC SYMPTOM**												
None	2 (66·7%)	2 (66·7%)	2 (66·7%)	2 (66·7%)	2 (66·7%)	1 (33·3%)	2 (40·0%)	2 (40·0%)	4 (80·0%)	3 (60·0%)	4 (80·0%)	5 (100%)
Mild	1 (33·3%)	1 (33·3%)	1 (33·3%)	0 (0%)	1 (33·3%)	2 (66·7%)	3 (60·0%)	3 (60·0%)	1 (20·0%)	2 (40·0%)	1 (20·0%)	0 (0%)
Moderate	0 (0%)	0 (0%)	0 (0%)	1 (33·3%)	0 (0%)	0 (0%)	0 (0%)	0 (0%)	0 (0%)	0 (0%)	0 (0%)	0 (0%)
Severe	0 (0%)	0 (0%)	0 (0%)	0 (0%)	0 (0%)	0 (0%)	0 (0%)	0 (0%)	0 (0%)	0 (0%)	0 (0%)	0 (0%)

For participants reporting a symptom on multiple days, the symptom is counted once at the maximal severity. The severity of solicited adverse events was graded mild, moderate, and severe. Data are n (%). IM denotes intramuscular, and SC subcutaneous

Table S3. Adverse events following vaccination with Trimer 4571.

Subject	Group	Related AE	Severity	Vaccine	Number of Administrations	Days since last Administration	Duration of AE
5	100 mcg SC	Pruritus at the injection site	Mild	Trimer 4571	2	3	6 days
	100 mcg SC	Pruritus at the injection site	Mild	Trimer 4571	3	0	9 days
6	100 mcg SC	Pruritus at the injection site	Mild	Trimer 4571	3	2	2 day
7	500 mcg IM	Pruritus at the injection site	Mild	Trimer 4571	2	2	1 day
	500 mcg IM	Pruritus at the injection site	Mild	Trimer 4571	3	2	1 day
8	500 mcg IM	Pruritus at the injection site	Mild	Trimer 4571	3	2	1 day
9	500 mcg IM	Neutropenia	Mild	Trimer 4571	2	7	8 days
12	500 mcg SC	Pruritus at the injection site	Mild	Trimer 4571	2	5	2 day
	500 mcg SC	Pruritus at the injection site	Mild	Trimer 4571	3	3	2 day
13	500 mcg SC	Pruritus at the injection site	Mild	Trimer 4571	2	6	1 day

Mild severity indicates no effect on activities of daily living, and moderate severity indicates some interference with activity not requiring medical intervention.

References

1. Cheng C, Duan H, Xu K, et al. Immune Monitoring Reveals Fusion Peptide Priming to Imprint Cross-Clade HIV-Neutralizing Responses with a Characteristic Early B Cell Signature. *Cell Rep* 2020; **32**(5): 107981.

2. Zhou T, Teng IT, Olia AS, et al. Structure-Based Design with Tag-Based Purification and In-Process Biotinylation Enable Streamlined Development of SARS-CoV-2 Spike Molecular Probes. *Cell Rep* 2020; **33**(4): 108322.

3. Kwon YD, Pancera M, Acharya P, et al. Crystal structure, conformational fixation and entry-related interactions of mature ligand-free HIV-1 Env. *Nat Struct Mol Biol* 2015; **22**(7): 522-31.

4. Jiang X, Burke V, Totrov M, et al. Conserved structural elements in the V3 crown of HIV-1 gp120. *Nat Struct Mol Biol* 2010; **17**(8): 955-61.

5. Stanfield RL, Gorny MK, Zolla-Pazner S, Wilson IA. Crystal structures of human immunodeficiency virus type 1 (HIV-1) neutralizing antibody 2219 in complex with three different V3 peptides reveal a new binding mode for HIV-1 cross-reactivity. *J Virol* 2006; **80**(12): 6093-105.

6. Gorny MK, Sampson J, Li H, et al. Human anti-V3 HIV-1 monoclonal antibodies encoded by the VH5-51/VL lambda genes define a conserved antigenic structure. *PLoS One* 2011; **6**(12): e27780.

7. Rini JM, Stanfield RL, Stura EA, Salinas PA, Profy AT, Wilson IA. Crystal structure of a human immunodeficiency virus type 1 neutralizing antibody, 50.1, in complex with its V3 loop peptide antigen. *Proc Natl Acad Sci U S A* 1993; **90**(13): 6325-9.

8. Cheng C, Xu K, Kong R, et al. Consistent elicitation of cross-clade HIV-neutralizing responses achieved in guinea pigs after fusion peptide priming by repetitive envelope trimer boosting. *PLoS One* 2019; **14**(4): e0215163.

9. Corrigan AR, Duan H, Cheng C, et al. Fusion peptide priming reduces immune responses to HIV-1 envelope trimer base. *Cell Rep* 2021; **35**(1): 108937.

10. Kong R, Xu K, Zhou T, et al. Fusion peptide of HIV-1 as a site of vulnerability to neutralizing antibody. *Science* 2016; **352**(6287): 828-33.

11. Cottrell CA, van Schooten J, Bowman CA, et al. Mapping the immunogenic landscape of near-native HIV-1 envelope trimers in non-human primates. *PLoS Pathog* 2020; **16**(8): e1008753.

12. Sarzotti-Kelsoe M, Bailer RT, Turk E, et al. Optimization and validation of the TZM-bl assay for standardized assessments of neutralizing antibodies against HIV-1. *J Immunol Methods* 2014; **409**: 131-46.

13. Decker JM, Bibollet-Ruche F, Wei X, et al. Antigenic conservation and immunogenicity of the HIV coreceptor binding site. *J Exp Med* 2005; **201**(9): 1407-19.

14. Punjani A, Rubinstein JL, Fleet DJ, Brubaker MA. cryoSPARC: algorithms for rapid unsupervised cryo-EM structure determination. *Nat Methods* 2017; **14**(3): 290-6.

15. Pettersen EF, Goddard TD, Huang CC, et al. UCSF Chimera--a visualization system for exploratory research and analysis. *J Comput Chem* 2004; **25**(13): 1605-12.

Official Title: A Phase I Dose Escalation, Randomized, Open-Label Clinical Trial to Evaluate Dose, Safety, Tolerability and Immunogenicity of a HIV-1 Vaccine, VRC-HIVRGP096-00-VP, With Alum in Healthy Adults

ClinicalTrials.gov Identifier: NCT03783130

Document Type:

- Redacted Final Version of the Study Protocol (Statistical Analysis Considerations located in Section 6 of the Protocol)
- Redacted Final Version of IRB-approved Informed Consent

Document/Date:

- Protocol Version 3.0: September 3, 2019
- Version 3.0 IRB-approved Informed Consent (IRB approval: September 30, 2019)

Version 3.0
September 3, 2019

VACCINE RESEARCH CENTER

Protocol VRC 018
(NIH 19-I-0031)

A PHASE 1 DOSE ESCALATION, RANDOMIZED, OPEN-LABEL CLINICAL TRIAL TO EVALUATE DOSE, SAFETY, TOLERABILITY AND IMMUNOGENICITY OF A HIV-1 VACCINE, VRC-HIVRGP096-00-VP, WITH ALUM IN HEALTHY ADULTS

Study Agent Provided by:
Vaccine Research Center (VRC),
National Institute of Allergy and Infectious Diseases (NIAID)
National Institutes of Health (NIH),
Bethesda, MD

Clinical Trial Sponsored by:
Vaccine Research Center (VRC), NIAID, NIH
Bethesda, MD

IND Sponsored by:
Vaccine Research Center (VRC), NIAID, NIH
Bethesda, MD

IND 18602 - held by NIAID

Principal Investigator:
Martin Gaudinski, M.D.
VRC, NIAID, NIH
Bethesda, MD 20892

IRB Initial Review Date: November 05, 2018

TABLE OF CONTENTS

Page

PRÉCIS **7**

1. INTRODUCTION **8**

1.1 Rationale for the Evaluation of VRC-HIVRGP096-00-VP (Trimer 4571) 8
1.2 Rationale for Use of Aluminum-Based Adjuvant 9
1.3 Assessment of immunogenicity 10

2. STUDY PRODUCTS **11**

2.1 VRC-HIVRGP096-00-VP, Investigational Vaccine 11
2.2 Aluminum Hydroxide Suspension, Adjuvant 11
2.3 VRC-PBSPLA043-00-VP, Diluent 11
2.4 Previous Human Experience 11

3. STUDY OBJECTIVES **12**

3.1 Primary Objectives 12
3.2 Secondary Objectives 12
3.3 Exploratory Objectives 12

4. STUDY DESIGN AND CLINICAL PROCEDURES **13**

4.1 Study Population 13
4.1.1 Inclusion Criteria 13
4.1.2 Exclusion Criteria 14
4.2 Clinical Procedures and Evaluations 15
4.2.1 Screening 15
4.2.2 Enrollment, Study Days and Visit Numbers 15
4.2.3 Product Administration 16
4.2.4 Clinical Follow-up and Solicited Adverse Events 16
4.2.5 Concomitant Medications 17
4.3 Dose Escalation Plan 17
4.4 Monitoring for HIV Infection 17
4.5 Criteria for Discontinuation of Product Administration or Subject Participation 18
4.5.1 Discontinuation from Product Administrations 18
4.5.2 Discontinuation from Protocol Participation 18
4.6 Pausing and Resuming the Study 19
4.6.1 Criteria for Pausing the Study 19
4.6.2 Plan for Review of Pauses and Resuming Rules 19

5. SAFETY AND ADVERSE EVENTS **20**

5.1 Adverse Events 20
5.2 Serious Adverse Events 20
5.3 Reporting SAEs to the IND Sponsor 21
5.4 IND Sponsor Reporting to the FDA 21
5.5 Reporting to the IRB 21
5.5.1 Unanticipated Problem 22

5.5.2 Non-Compliance ... *22*
5.5.3 Protocol Deviation ... *23*
5.5.4 Death ... *23*
5.5.5 New Information ... *23*
5.5.6 Suspension or Termination of Research Activities ... *24*
5.5.7 Expedited Reporting to the IRB ... *24*
5.5.8 Annual Reporting to the IRB ... *24*

6. STATISTICAL CONSIDERATIONS ... 25

6.1 Overview ... 25
6.2 Objectives ... 25
6.3 Endpoints ... 25
6.3.1 Primary Endpoints: Safety ... *25*
6.3.2 Secondary Endpoints: Immunogenicity ... *25*
6.3.3 Exploratory Endpoints: Immunogenicity ... *25*
6.4 Sample Size and Accrual ... 25
6.4.1 Power Calculations for Safety ... *26*
6.5 Statistical Analysis ... 27
6.5.1 Baseline Demographics ... *27*
6.5.2 Safety Analysis ... *27*
6.5.3 Analysis of Immune Responses ... *28*
6.5.4 Missing Data ... *28*
6.5.5 Randomization of Treatment Assignments ... *28*

7. STUDY PRODUCTS AND PHARMACY PROCEDURES ... 29

7.1 Study Products ... 29
7.2 Study Product Labels ... 29
7.3 Study Product Storage ... 29
7.3.1 Temperature Excursions ... *30*
7.4 Preparation of Study Product-Adjuvant for IM and SC Administration ... 30
7.4.1 Preparation of the 500 mcg Dose with Adjuvant ... *30*
7.4.2 Preparation of the 100 mcg Dose with Adjuvant ... *31*
7.5 Study Product Accountability ... 31
7.5.1 Documentation ... *31*
7.5.2 Disposition ... *31*

8. HUMAN SUBJECT PROTECTIONS AND ETHICAL OBLIGATIONS ... 32

8.1 Institutional review board ... 32
8.2 Subject recruitment and enrollment ... 32
8.2.1 Participation of Children ... *32*
8.2.2 Participation of Site Employees ... *32*
8.3 Informed Consent ... 32
8.4 Subject confidentiality ... 33
8.5 Risks and Benefits ... 33
8.5.1 Risks ... *33*
8.5.2 Benefits ... *34*
8.6 Plan for Use and Storage of Biological Samples ... 34

8.6.1 Use of Samples, Specimens and Data ... *34*
8.6.2 Storage and Tracking of Blood Samples and Other Specimens ... *34*
8.6.3 Disposition of Samples, Specimens and Data at Completion of the Protocol ... *35*
8.6.4 Loss or Destruction of Samples, Specimens or Data ... *35*
8.7 Compensation ... 35
8.8 Safety Monitoring ... 35
8.8.1 Protocol Safety Review Team (PSRT) ... *35*

9. ADMINISTRATIVE AND LEGAL OBLIGATIONS ... 36

9.1 Protocol Amendments and Study Termination ... 36
9.2 Study Documentation and Study Records Retention ... 36
9.3 Data Collection, Data Sharing, and Protocol Monitoring ... 36
9.3.1 Data Collection ... *36*
9.3.2 Data Sharing ... *36*
9.3.3 Source Documents ... *37*
9.3.4 Protocol Monitoring ... *37*
9.4 Language ... 37
9.5 Policy Regarding Research-Related Injuries ... 37

10. REFERENCES ... 38

APPENDIX I: CONTACT INFORMATION ... 40

APPENDIX II: SCHEDULE OF EVALUATIONS ... 42

APPENDIX III: ASSESSING RELATIONSHIP AND GRADING SEVERITY OF ADVERSE EVENTS ... 47

ABBREVIATIONS

Abbreviation	Term
AE	adverse event
AIDS	acquired immunodeficiency syndrome
ALT	alanine aminotransferase
AoU	assessment of Understanding
ART	Antiretroviral therapy
AUC	area under the curve
BMI	body mass index
bNAbs	broadly neutralizing antibodies
CBC	complete blood count
CDs	coding sequences
CL	clearance
cGMP	current Good Manufacturing Practice
DAIDS	Division of AIDS
DHHS	Department Health & Human Services
DNA	deoxyribonucleic acid
DSMB	Data and Safety Monitoring Board
EC50	half-maximal effective concentration
EDTA	Ethylenediaminetetraacetate
ELICA	Electrochemiluminescence
ELISA	enzyme-linked immunosorbent assay
EM	Negative stain electron microscopy
Env	envelope
FDA	Food and Drug Administration
GCP	Good Clinical Practice
GLP	Good Laboratory Practice
GLT	(green) lithium heparin tube
HIV	human immunodeficiency virus
HLA	human leukocyte antigen
HRPP	Human Research Protections Program
IB	Investigator's Brochure
IC	Institute/Center
ICF	Informed Consent Form
IgG1	Immunoglobulin G1
IM	Intramuscular
IND	investigational new drug application
IRB	Institutional Review Board
ISM	Independent Safety Monitor
ITC	Isothermal Calorimetry
kg	kilogram
L	liter
LIMS	Laboratory Information Management System

Abbreviation	Term
LLN	Lower limit of normal
λz	terminal slope of concentration vs time profile
mcg	microgram
MedDRA	medical dictionary for regulatory activities
mg	milligram
mL	milliliter
mM, mmol	millimole
MO	Medical Officer
MPER	Membrane proximal external region
MSD	Meso Scale Discovery
NHP	non-human primate
NIAID	National Institute of Allergy and Infectious Diseases
NIH	National Institutes of Health
NIH CC	National Institutes of Health Clinical Center
OHRP	Office for Human Research Protections
PBMC	peripheral blood mononuclear cells
PBS	phosphate buffered saline
PCR	polymerase chain reaction
PI	Principal Investigator
PK	pharmacokinetic
PSRT	Protocol Safety Review Team
Q	inter-compartmental clearance
QA	quality assurance
SAE	serious adverse event
SC	subcutaneous
SHIV	simian-human immunodeficiency virus
SOE	schedule of evaluations
SST	serum separator tube
TCR	tissue cross reactivity
T/F	transmitted/founder
$T_{1/2}$	half-life
Tmax	time of maximal concentration (Cmax)
ULN	Upper limit of normal
UP	unanticipated problem
VCMP	Vaccine Clinical Materials Program
VIP	Vaccine Immunology Program
VITL	Vaccine Immunology Testing Laboratory
VRC	Vaccine Research Center
WBC	white blood cell

PRÉCIS

Title: **VRC 018**: A Phase I Dose Escalation, Randomized, Open-Label Clinical Trial to Evaluate Dose, Safety, Tolerability and Immunogenicity of a HIV-1 Vaccine, VRC-HIVRGP096-00-VP, with Alum in Healthy Adults

Design: This is a Phase I, open-label, dose escalation study to evaluate the dose, safety, tolerability, and immunogenicity of VRC-HIVRGP096-00-VP (Trimer 4571) with aluminum hydroxide suspension (alum) as adjuvant in a three-injection regimen. The hypotheses are that the vaccine will be safe and tolerable and will induce detectable immune responses. The primary objective is to evaluate the safety and tolerability of the investigational vaccine at three doses administered with alum. Secondary objectives are to evaluate humoral and cellular immunogenicity of the investigational vaccine regimens.

Study Products: VRC-HIVRGP096-00-VP (Trimer 4571) was developed by the Vaccine Research Center (VRC), National Institute of Allergy and Infectious Diseases (NIAID). The soluble HIV-1 envelope product consists of an HIV-1 envelope (Env) trimer variant, derived from clade A, strain BG505, with stabilizing mutations and engineered disulfide bonds, specifically recognized by broadly neutralizing antibodies and resists gp120 conformational change caused by CD4 binding. Injections will be administered intramuscularly (IM) and subcutaneously (SC) in a 1 mL volume by needle and syringe. The product is provided at a 500 mcg/mL concentration in 3 mL glass vials filled to 1.2 ± 0.10 mL.

Adjuvant is an aluminum hydroxide suspension (alum) provided in a sterile, pyrogen-free suspension at a concentration of 5 mg/mL in 3 mL glass vials filled to 0.7 ± 0.10 mL.

Subjects: Healthy adults ages 18 to 50.

Study Plan: Subjects will receive VRC-HIVRGP096-00-VP at doses of 100 mcg or 500 mcg, both with 500 mcg alum field mixed, administered via IM or SC injections. A dose escalation evaluation will occur to ensure the safety data support proceeding to the higher dose. Subjects will be evaluated for safety and immune responses through blood collection at specified timepoints throughout the study. The study schema is below:

**VRC 018 Study Schema**						
**Group**	**Subjects**	**Route**	**Dose** (mcg)	**Day 0**[1]	**Week 8**[1]	**Week 20**[1]
1	3	IM	100	X	X	X
2	3	SC	100	X	X	X
3	5	IM	500	X	X	X
4	5	SC	500	X	X	X
**Total**	*16	[1]500 mcg of alum will be field mixed with Trimer 4571 for all groups<br>*Up to 25 subjects may be enrolled if needed to evaluate safety or immunogenicity.				

Duration: Subjects will be followed for 40 weeks.

1. INTRODUCTION

In the nearly four decades since acquired immunodeficiency syndrome (AIDS) was first observed and described, its virologic etiologic agent, human immunodeficiency virus (HIV) has continued to be an important worldwide threat to public health [1-7]. In 2017 in the United States alone, an estimated 1.8 million new infections and 940,000 AIDS-related deaths occurred [8]. Global statistics reported in 2017 are even more sobering, with approximately 36.9 million people worldwide living with HIV[8].

For individuals in the 1980s and early 1990s who received an HIV diagnosis, median survival was less than a year [9, 10]. Advances in therapeutic drugs and increased access to combination antiretroviral therapy (ART) have transformed the survival prognosis for newly-diagnosed individuals in 2017 to one of an essentially normal life span [11, 12]. However, new infections have not declined significantly and remain at approximately 2 million cases each year. Development of a protective HIV vaccine thus remains a global health priority to control and eliminate the AIDS pandemic [13].

A central goal of HIV-1 vaccine development is to elicit neutralizing antibodies against diverse tier 2 (neutralization-resistant) viral isolates that represent naturally circulating HIV-1 strains. A primary vaccine epitope has been the HIV-1 envelope glycoprotein (Env) which is the sole viral component exposed on the virion surface and therefore the principal target for neutralizing antibodies. In a viral infection, HIV Env is first synthesized as a precursor glycoprotein, gp160, and then cleaved to gp120 receptor-binding and gp41 transmembrane subunits, and further assembled into a trimer of gp120-gp41 heterodimers via noncovalent interactions [14, 15]. To infect a target cell, the gp120 subunit binds to the host CD4 receptor, triggering conformational rearrangements in HIV Env to allow binding to CCR5 or CXCR4. This co-receptor interaction causes the gp41 fusion peptide to insert into the host cell membrane inducing fusion of virion and host cell membranes [16, 17]. Extensive glycosylation, sequence variation and conformational masking of key neutralizing epitopes allow HIV Env to evade the host immune response thus creating unique challenges for vaccine developers. Early vaccine trials using uncleaved trimer mimics or gp120-based immunogens with exposed surface loops and non-neutralizing epitopes were unsuccessful in eliciting a tier 2 neutralizing response [18]. Structural studies revealed that pre-fusion Env on infectious virions undergoes transitions among at least three distinct conformations [19-21]. Most HIV-1 broadly neutralizing antibodies (bNAb) preferentially target a prefusion closed conformation of HIV Env trimer [20-23]. A major goal for vaccine developers has therefore been to produce an HIV-1 Env trimer stabilized in this prefusion closed conformation in order to present bNAb producing epitopes and also mask immunodominant but non-neutralizing epitopes which may also interfere with eliciting broadly neutralizing antibody responses.

1.1 Rationale for the Evaluation of VRC-HIVRGP096-00-VP (Trimer 4571)

The VRC, NIAID, NIH developed the VRC-HIVRGP096-00-VP vaccine (Trimer 4571) candidate, which mimics the native HIV-1 Env complex. Trimer 4571 was derived from the clade A, strain BG505 Env gene, and stabilized with engineered disulfide bonds and a truncated residue 664 in gp41 to produce a soluble, cleaved Env trimer in a prefusion closed conformation [21, 23]. A 201C 433C disulfide mutation introduced within the gp120 subunit maintains the Env trimer in a closed state that is resistant to CD4-induced conformational change [21]. Trimer 4571 exhibits the desired antigenic profile which is specifically recognized by broadly neutralizing antibodies rather than by poorly neutralizing V3-directed and CD4-induced antibodies, even in the presence of CD4 [21, 24].

To evaluate the immunogenicity of Trimer 4571, guinea pigs received 50 mcg of study product with or without adjuvant by needle and syringe intramuscularly (IM) at 0, 4, and 8 weeks. Trimer 4571 elicited specific responses as measured by ELISA and were detected after each immunization. After the first immunization, higher neutralization titers were observed for the adjuvanted group compared to the unadjuvanted group. After three immunizations, the adjuvanted group showed significantly higher neutralization titers compared to the unadjuvanted group.

Translatability of neutralizing activity was evaluated in the non-human primate (NHP) model. Rhesus macaques were immunized with 100 mcg of adjuvanted Trimer 4571 by needle and syringe IM according to the same 0, 4, and 16-week schedule as administered to the guinea pigs. Similarly, trimer specific responses were detected after two immunizations with an increased response after the third immunization for each group.

Immune response elicited by NHP following immunization with adjuvanted Trimer 4571 administered subcutaneously (SC) were similar to results observed previously with adjuvanted Trimer 4571 administered IM.

Preclinical studies suggest that using unadjuvanted Trimer 4571 is not immunogenic. Therefore, groups of Trimer 4571 without adjuvant are not being evaluated in this study.

Based on its safety and immunogenicity profiles in preclinical studies, the candidate Trimer 4571 vaccine with adjuvant is being evaluated for the first time in healthy adults in this Phase 1 clinical trial. Vaccine regimens and doses used in this study were based on pre-clinical studies of HIV-1 Trimer 4571 in guinea pigs and rhesus macaques and previous experience with VRC phase 1 HIV vaccine studies.

1.2 Rationale for Use of Aluminum-Based Adjuvant

Adjuvants improve the elicited immune response to many vaccines [25, 26]. The most common aluminum-based adjuvants are aluminum hydroxide, aluminum phosphate, potassium aluminum sulfate, or mixed aluminum salts [27, 28]. Several phenomena contribute to the effect of aluminum hydroxide based adjuvants including: 1) a ‘repository effect’ that occurs when the antigens aggregate on the adjuvant particle and are deposited to the immune cell for long duration to induce immune responses, 2) a pro-phagocytic effect, and 3) the possible activation of the pro-inflammatory nucleotide-like receptor protein dependent pathways [28]. Aluminum-based adjuvants often improve humoral and innate responses and may lead to increased antibody titers, rapid induction of responses, reduction in size or frequency of doses, increased breadth of responses to overcome pathogen diversity, induction of long-lasting immune memory responses, and induction of response to overcome poor immune systems in elderly and young children [29-32]. Based on over six decades of use, aluminum is broadly accepted to be safe, well-tolerated and effective [29, 30].

As per 21 Code of Federal Regulations (CFR) part 610.15, the amount of aluminum in biological products cannot exceed 0.85 mg/dose. The amount of aluminum in vaccines currently licensed in the US ranges from 0.125-0.85 mg/dose [33]. Based on FDA regulations, experience with HPV VLP vaccines and VRC pre-clinical data, 0.5 mg/dose of aluminum hydroxide adjuvant will be used in this phase 1 study.

1.3 Assessment of immunogenicity

The primary immunogenicity time point is two weeks after the third dose. Specimens to evaluate immunogenicity will be collected at baseline and at specified time points throughout the study after the administration of product. HIV-specific humoral immune responses will be assessed by neutralization antibody assays.

Trimer 4571-specfic antibody titers will be measured by Electrochemiluminescence (ELICA) using a Meso Scale Discovery (MSD) platform. Serum from the volunteers will be diluted 8-fold into 384 well plates first coated with streptavidin and subsequently coated with a biotinylated BG505 SOSIP DS-10ln protein. Trimer 4571-specfic antibodies bound to the BG505 will be detected using a SULFO-TAG secondary detection antibody. Area under the curve (AUC) will be calculated for each sample tested from the 8-fold serial dilution. AUC cutoff is defined from screening naïve samples and only a signal above the assay cutoff is considered as true positive for Trimer 4571-specific antibodies. Other exploratory assays to assess humoral and cellular immune responses may be performed with stored samples.

Human leukocyte antigen (HLA) type may be obtained from stored samples if needed to assess HLA-class restricted cellular responses. Additional measurements of antibody, B cell and T cell responses may also be assessed from stored samples for timepoints throughout the study as exploratory evaluations. This includes a number of high-throughput functional assays and high-throughput biophysical profiling tools to comprehensively characterize the humoral immune response elicited by vaccination with Trimer 4571.

The immunogenicity testing will be performed at the VRC's Vaccine Immunology Program (VIP), Antibody Analytics (AA) laboratory at the VRC, formerly Vaccine Immunology Testing Laboratory (VITL) or by other approved scientific collaborators.

2. STUDY PRODUCTS

Study products are manufactured under current Good Manufacturing Practice (cGMP) regulations by VRC/NIAID/NIH at the VRC Production Plant operated under contract by the Vaccine Clinical Materials Program (VCMP), Leidos Biomedical Research, Inc., Frederick, MD. Specific manufacturing information is included on the product vial and in the Investigator's Brochure (IB). Quality Assurance lot release testing by the manufacturer and ongoing stability programs verify conformance to product specifications throughout use in clinical trials

2.1 VRC-HIVRGP096-00-VP, Investigational Vaccine

The VRC-HIVRGP096-00-VP vaccine is a sterile, aqueous, buffered solution filled into single dose vials. Details on preclinical studies conducted with VRC-HIVRGP096-00-VP, as well as composition and manufacturing are available in the IB.

2.2 Aluminum Hydroxide Suspension, Adjuvant

The adjuvant in this study is aluminum hydroxide suspension (alum), a sterile, pyrogen-free, suspension.

2.3 VRC-PBSPLA043-00-VP, Diluent

The diluent is composed of phosphate buffered saline (PBS) aseptically filled into single dose vials.

2.4 Previous Human Experience

There is no human experience with VRC-HIVRGP096-00-VP alone or with adjuvant.

Aluminum adjuvants have been used in vaccines for many decades with a demonstrated safety profile [34]. Aluminum is the most common adjuvant used in human vaccines licensed by the Food and Drug Administration (FDA) [33, 35]. Aluminum-containing vaccines have been associated with erythema, subcutaneous (SC) nodules, contact hypersensitivity, granulomatous inflammation [34, 36].

Aluminum-based adjuvants promote strong humoral immune responses, and therefore, are incorporated in vaccines against diseases where neutralizing antibodies are required for protection such as diphtheria, tetanus and hepatitis B. The licensed HPV VLP vaccines, Gardasil (Merck & Co, Inc.) and Cervarix (GlaxoSmithKline) have aluminum in the formulations at 0.5 mg of aluminum hydroxyphosphate sulphate per dose and 0.5 mg of aluminum hydroxide per dose, respectively [37].

3. STUDY OBJECTIVES

3.1 Primary Objectives

- To evaluate the safety and tolerability of adjuvanted Trimer 4571 administered IM or SC at a dose of 100 mcg to healthy adults
- To evaluate the safety and tolerability of adjuvanted Trimer 4571 administered IM or SC at a dose of 500 mcg to healthy adults

3.2 Secondary Objectives

- To evaluate the ELICA titer response to adjuvanted Trimer 4571 at two weeks after the third dose

3.3 Exploratory Objectives

- To explore and characterize the humoral and cellular response to adjuvanted Trimer 4571

4. STUDY DESIGN AND CLINICAL PROCEDURES

This is an open-label, randomized, dose escalation study to examine the safety, tolerability and dose of the Trimer 4571 vaccine with adjuvant in healthy adults. The hypotheses are that the vaccine will be safe and tolerable for human administration and will induce detectable immune responses.

The study schema is shown in **Table 1**.

Table 1: VRC 018 Study Schema						
**Group**	**Subjects**	**Route**	**Dose** (mcg)	**Day 0**[1]	**Week 8**[1]	**Week 20**[1]
1	3	IM	100	X	X	X
2	3	SC	100	X	X	X
3	5	IM	500	X	X	X
4	5	SC	500	X	X	X
**Total**	*16	[1]500 mcg of alum will be field mixed with Trimer 4571 for all groups<br>*Up to 25 subjects may be enrolled if needed to evaluate safety or immunogenicity.				

The study will be conducted at the VRC Vaccine Evaluation Clinic (VEC) in the NIH Clinical Center (NIH CC). The expected duration of time on study is approximately 40 weeks.

4.1 Study Population

All inclusion and exclusion criteria must be met for eligibility.

4.1.1 Inclusion Criteria

A subject must meet all of the following criteria:

1. Able and willing to complete the informed consent process.
2. 18-50 years old, inclusive, on day of enrollment.
3. Available for clinic follow-up through the last study visit.
4. Able to provide proof of identity to the satisfaction of the study clinician completing the enrollment process.
5. Willing to donate blood for sample storage to be used for future research.
6. In good general health without clinically significant medical history.
7. Physical examination and laboratory results without clinically significant findings.
8. Body Mass Index (BMI) $\leq$ 40.
9. Assessed as low risk for HIV acquisition by agreeing to discuss HIV infection risks, agreeing to risk reduction counseling, and agreeing to avoid behavior associated with high risk of HIV exposure through the end of study.
10. Screening laboratory values within 56 days prior to enrollment that meet the following criteria:
    - Hemoglobin within the institutional normal limits

- White blood cell (WBC) count between 2,500-12,000/mm3
- WBC differential absolute cell counts either within institutional normal range or accompanied by site PI or Associate Investigator (AI) approval, except neutrophils and lymphocytes must specifically be within the range of ≥0.75 x the lower limit of normal (LLN) and ≤1.25 x the upper limit of normal (ULN) for neutrophil and lymphocyte absolute counts
- Platelets = 125,000-500,000/mm^3
- Alanine aminotransferase (ALT) ≤ 1.25 x ULN based on the institutional normal range
- Serum creatinine ≤ 1.1 x ULN based on the institutional normal range
- Negative for HIV infection by an FDA approved method of detection

Woman-specific (if presumed to be of childbearing potential):

11. Agrees to use effective means of birth control from at least 21 days prior to enrollment through the end of the study.
12. Negative β-HCG (human chorionic gonadotropin) pregnancy test (urine or serum) on day of enrollment.

4.1.2 Exclusion Criteria

A subject will be excluded if one or more of the following conditions apply:

Woman-specific:

1. Breast-feeding or planning to become pregnant through the end of study.

Subject has received any of the following:

2. An investigational HIV vaccine.
3. Systemic glucocorticoid use equal or greater than prednisone 20mg/day within 4 weeks prior to enrollment, or other medication use likely to impair vaccine response.
4. Blood products within 16 weeks prior to enrollment.
5. Live attenuated vaccines within 4 weeks prior to enrollment.
6. Inactivated vaccines within 2 weeks prior to enrollment.
7. Investigational research agents within 4 weeks prior to enrollment.
8. Current allergen immunotherapy with antigen injections, unless on maintenance schedule.
9. Current anti-TB prophylaxis or therapy.

Subject has any of the following:

10. Serious reactions to vaccines that preclude receipt of study injections as determined by the principal investigator or designee.
11. Hereditary angioedema, acquired angioedema, or idiopathic forms of angioedema.
12. Hypertension that is not well controlled.

13. Evidence of significant autoimmune disease or immunodeficiency.
14. Idiopathic urticaria within the past year.
15. Bleeding disorder diagnosed by a doctor (e.g. factor deficiency, coagulopathy, or platelet disorder requiring special precautions) or significant bruising or bleeding difficulties with IM injections or blood draws.
16. Seizure disorder other than: 1) febrile seizures, 2) seizures secondary to alcohol withdrawal more than 3 years ago, or 3) seizures that have not required treatment within the last 3 years.
17. Asplenia or functional asplenia.
18. Any other chronic or clinically significant condition that in the opinion of the investigator would jeopardize the safety or rights of the study subject including but not limited to: diabetes mellitus type I, chronic hepatitis; OR clinically significant forms of: drug or alcohol abuse, asthma, psychiatric disorders, heart disease, or cancer.

4.2 Clinical Procedures and Evaluations

Evaluation of this investigational vaccine will include laboratory tests, medical history, physical assessment by clinicians and subject self-assessment. The schedule of study visits is shown in the Schedule of Evaluations (SOE, **Appendix II**). Total blood volume drawn from each subject will comply with NIH CC Guidelines.

4.2.1 Screening

Screening for this study will be completed through the VRC's screening protocol, VRC 500 (NIH 11-I-0164). Subjects will be recruited through Institutional Review Board (IRB)-approved advertising. Screening evaluations and sample collection include medical history review, physical exam, and any clinical laboratory tests as detailed in the SOE (**Appendix II**) needed to confirm eligibility. Women presumed to be of reproductive potential will be given a pregnancy test. Additional assessments of health may be conducted at screening based on clinical judgment. All blood samples for research purposes only as shown in the SOE will be collected during screening, however these samples can be obtained at any time during screening, regardless of the 56-day window prior to enrollment.

Informed consent documents will be reviewed during screening. Counseling related to potential risks of the study product, pregnancy prevention and HIV risk-reduction will be performed. An Assessment of Understanding (AoU) will be completed in association with enrollment into VRC 018. Records will be kept to document the reason that screened subjects did not enroll.

4.2.2 Enrollment, Study Days and Visit Numbers

In this study, enrollment is defined as the assignment of a study identification number and a study group to a subject in the clinical database. A clinician will discuss the target dates and timing of the study product administration(s) and sample collections before completing an enrollment to help ensure that the subject can comply with the projected schedule.

Day 0 is defined as the day of protocol enrollment and first product administration. Medical history and Day 0 evaluations prior to the first product administration are the baseline for subsequent safety assessments.

4.2.3 Product Administration

All product administrations will be completed according to the assigned group. On the day of and prior to each product administration, vital signs (temperature, blood pressure, and heart rate (pulse) will be recorded, a targeted physical examination may be conducted as needed, and women of childbearing potential must have a negative pregnancy test.

All IM injections will be administered into the deltoid muscle by needle and syringe. All SC injections will be administered into the SC tissue in the posterior upper arm.

When choosing a site for injection, clinicians should consider whether there is an arm injury, local skin problem or significant tattoo that precludes administering the injection or that will interfere with evaluating the arm after injection.

Prior to product administration, invert the syringe 5x to mix the study product. After preparation in syringes for IM or SC administration, the prepared product may be stored at 2ºC to 8ºC for up to 24 hours and/or at controlled room temperature (15°C to 27ºC) for up to 4 hours, including administration time. The product may not be stored in direct sunlight.

It is recommended but not required that the first injection be administered into a non-dominant arm and that subsequent injections be given in alternate arms.

Procedures for preparation of Trimer 4571 are described in **Section 7**.

4.2.4 Clinical Follow-up and Solicited Adverse Events

Post-Product Administration Follow-Up: Following each product administration, subjects will be observed for a minimum of 30 minutes. Prior to discharge from the clinic and at least 30 minutes post-injection, vital signs will be recorded, and the injection site will be inspected for evidence of local reaction. Subjects will be contacted by telephone on the day after each study injection as shown on the Schedule of Evaluations (**Appendix II**).

Solicited Adverse Events (Reactogenicity): Subjects will be given a 7-day paper diary and access to an electronic-based diary, a thermometer, and a measuring device. The subject will use the diary to record daily their highest temperature, local and systemic symptoms, and concomitant medications for 7 days. Subjects will be provided training on diary completion, proper thermometer usage, and the use of the measuring device to measure any injection site bruising, swelling and/or redness. Completion of diary training will be noted in the source documents. Note that subjects will be encouraged to use the preferred electronic diary but will have the option to use a paper diary. The paper diary if used, will be transcribed into the study database and stored in the subject file for monitoring purposes.

The signs and symptoms solicited by the diary will include systemic events of feeling unusually tired or unwell, muscles aches (other than at injection site), headache, chills, nausea, and local events at the injection site including pain/tenderness, bruising, swelling and redness. Subject diaries will be reviewed by a clinician for accuracy and completeness at follow-up visits. No attribution assessment will be performed for solicited events reported in the diary. Clinicians will follow and collect resolution information for any solicited symptoms that have not resolved within 7 days.

Diary data will be available in real-time for subjects who use the electronic diary. The clinician may contact the subject by phone if any moderate or severe side effect is reported. Events that may require a clinic visit include rash, urticaria, fever of 38.6˚C (Grade 2) or higher lasting greater than

24 hours or significant impairment in the activities of daily living (ADL) (such as those consistent with Grade 2 or higher impairment). Additionally, other clinical concerns may prompt a study visit based on the judgment of a study clinician.

Clinical Follow-Up: Study follow-up will continue via clinical visits through 40 weeks after the first study injection. The visit schedule is based on intervals of time after each study product administration. In the event that a subject only receives one or two study vaccinations, alternate study schedules are provided in **Appendix II**.

The schedule of visits, allowable windows for completing the visits, and evaluations performed at each visit are shown in the SOE in **Appendix II**. After Day 0, deviations from the visit windows in completing study visits are discouraged and will be recorded as protocol deviations but are permitted at the discretion of the PI.

4.2.5 Concomitant Medications

Only routine prescription medications will be entered in the database at the time of enrollment. Subsequently, concomitant medications are only updated or recorded in the study database if there is an occurrence of an adverse event (AE) that requires expedited reporting or if the subject develops a new chronic condition that requires ongoing medical management. Otherwise, the concomitant medication changes throughout the study will be recorded in the subject's chart as needed for general medical records but will not be recorded in the study database.

4.3 Dose Escalation Plan

The study will begin with enrollment of subjects into Groups 1 and 2 in a 1:1 block randomization scheme. No further subjects will receive product until the first enrolled subject has completed Visit 02A and reports no safety concerns.

An interim safety review of all available data will occur when all subjects in Groups 1 and 2 who received study product have completed their "Study Week 2" visit. If assessed as safe to proceed by the Protocol Safety Review Team (PSRT), Groups 3 and 4 may begin enrollment at the higher dose.

If the first study injection is not completed or there are discontinuations from the study before there are sufficient data to conduct the dose escalation review for a group, then extra subjects may be enrolled directly into that group in order to have the requisite data on at least 6 subjects. Additionally, AEs assessed as related to the study product at the time of a dose escalation review may be judged by the PSRT to warrant adding additional subjects at a given dose level.

The IRB will be provided with documentation of the safety review process and notification of the dose escalation review. Consultation with the IRB and FDA, if needed, as per study pause criteria (**Section 4.5**) will occur if indicated by the review. One outcome of a dose escalation review may be to recommend evaluation of additional subjects at the current dose level and reassess for safety before proceeding to a higher dose level.

4.4 Monitoring for HIV Infection

Although the study vaccine will not cause HIV infection, it may induce antibodies that are detected by standard HIV infection screening techniques. This is referred to as vaccine induced

seropositivity (VISP). The following steps will be taken to ensure detection of HIV infection and to protect participants from adverse consequences associated with VISP:

- Study participants will receive regularly scheduled counseling regarding avoidance of HIV infection in accordance with the most recent Centers for Disease Control and Prevention HIV counseling guidelines.

- Study participants will be screened for HIV infection periodically throughout the study as noted on the Schedule of Evaluations (Appendix II).Participants will be promptly informed and counseled if they become HIV-infected during the study and referred for treatment.

- Participants who are found to have VISP will be counseled regarding their test results. They will also be offered letters of explanation regarding their VISP results that they can keep for their records and share with healthcare providers as needed. Wallet cards will be provided to all study subjects to remind them who to contact regarding any VISP concerns that may arise during and after study participation.

4.5 Criteria for Discontinuation of Product Administration or Subject Participation

Decisions to discontinue study injections or protocol participation will be made by the PI.

4.5.1 Discontinuation from Product Administrations

A subject will be discontinued from study injections for the following reasons:

- Pregnancy;
- Grade 3 AE assessed as related to study product (except Grade 3 solicited reactogenicity lasting less than 48 hours);
- Grade 4 AE assessed as related to study product;
- Immediate hypersensitivity reaction associated with study product;
- Intercurrent illness that is not expected to resolve before the next scheduled product administration;
- Treatment with systemic glucocorticoids (e.g., prednisone or other glucocorticoid) or other immunomodulators (other than nonsteroidal anti-inflammatory drugs [NSAIDs]), with the exception that study injection may continue per PI discretion if the next one occurs at least 2 weeks following completion of glucocorticoid treatment; or,
- The PI assesses that it is not in the best interest of the subject to continue receiving study product.
- Any SAE of any grade assessed as related to study vaccine

4.5.2 Discontinuation from Protocol Participation

A subject may be discontinued from protocol participation for the following reasons:

- Subject voluntarily withdraws;

- The IND Sponsor or regulatory authorities stop the study; or,
- The PI assesses that it is not in the best interest of the subject to continue participation in the study or that the subject's compliance with the study is not sufficient.

4.6 Pausing and Resuming the Study

4.6.1 Criteria for Pausing the Study

The PI and PSRT will closely monitor and analyze study data as they become available and will make determinations regarding the presence and severity of AEs. Enrollments and study product administration will be paused by the PI, and the IND Sponsor will be promptly notified, according to the following criteria:

- **One** (or more) subject experiences a **Serious Adverse Event** (SAE) that is assessed as related to study product, or
- **Two** (or more) subjects experience the same **Grade 3 or higher unsolicited AE** assessed as related to study product.
- **Three** (or more) subjects experience a **Grade 3 or higher solicited AE** assessed as related to study product.

4.6.2 Plan for Review of Pauses and Resuming Rules

In the event of a pause, the IND Sponsor Medical Officer (MO) and the PSRT will be promptly notified. The IND Sponsor MO and PI, in consultation with the PSRT, will conduct a review of available information, including the events that lead to the pause, and will make the decision to resume, amend or close the study. As part of the pause review, the reviewers may also advise on whether the study needs to be paused again for any subsequent events of the same type. The FDA and IRB will be notified as needed of any changes to study status and of the review decisions. Study product administration and enrollments would resume only if review of the AEs that caused the pause results in a recommendation to permit further study product administrations and study enrollments.

5. SAFETY AND ADVERSE EVENTS

5.1 Adverse Events

An adverse event (AE) is any untoward or unfavorable medical occurrence in a human subject, including any abnormal sign (e.g., abnormal physical exam or laboratory finding), symptom, or disease temporarily associated with the subject's participation in research, whether or not considered related to the subject's participation in the research. In the context of FDA-required reporting, an AE means any untoward medical occurrence associated with the use of a drug in humans, whether or not considered drug related.

Appendix III describes how the relationship between an AE and the study product will be assessed. Also available in Appendix III is the link to the Division of AIDS (DAIDS) Table for Grading the Severity of Adult and Pediatric Adverse Events, Corrected Version 2.1 (July 2017), which will be used to determine the severity grades of AEs in this protocol. The following guidelines will be used to determine if an AE should be recorded in the database:

- Solicited AEs (i.e. reactogenicity parameters) will be collected through the daily diary and recorded in the study database for 7 days after each study injection. Clinicians will follow and collect resolution information for any reactogenicity symptoms that are not resolved within 7 days.
- Unsolicited AEs will be recorded in the study database from the date of receipt of each study injection through completion of the visit scheduled at 28 days after each injection with the collection of product attribution assessments. At other time periods between injections and when greater than the "28-day" post injection visit, only SAEs (as detailed in **Section 5.2**) and new chronic medical conditions will be recorded as AEs in the database through the last study visit.

5.2 Serious Adverse Events

The term "Serious Adverse Event" (SAE) is defined in 21 CFR 312.32 as follows: "An adverse event or suspected adverse reaction is considered 'serious' if, in the view of either the investigator or the sponsor, it results in any of the following outcomes: Death, a life-threatening adverse event, inpatient hospitalization or prolongation of existing hospitalization, a persistent or significant incapacity or substantial disruption of the ability to conduct normal life functions, or a congenital anomaly/birth defect. Important medical events that may not result in death, be life-threatening, or require hospitalization may be considered serious when, based upon appropriate medical judgment, they may jeopardize the patient or subject and may require medical or surgical intervention to prevent one of the outcomes listed in this definition. Examples of such medical events include allergic bronchospasm requiring intensive treatment in an emergency room or at home, blood dyscrasias or convulsions that do not result in inpatient hospitalization, or the development of drug dependency or drug abuse."

"Life-threatening" refers to an adverse event that at occurrence represents an immediate risk of death to the subject. An event that may cause death if it occurs in a more severe form is not considered life-threatening. Similarly, a hospital admission for an elective procedure is not considered a SAE.

5.3 Reporting SAEs to the IND Sponsor

AEs that meet SAE criteria per the FDA definition (Section 5.2) must be reported and submitted by the clinical site on an expedited basis to the IND Sponsor, VRC/NIAID/NIH, according to sponsor guidelines as follows:

- results in death;
- is life-threatening;
- results in persistent or significant disability/incapacity;
- requires unplanned inpatient hospitalization or prolongation of existing hospitalization;
- is a congenital anomaly/birth defect in the offspring of a study subject; or,
- is an important medical event that may jeopardize the subject or may require intervention to prevent one of the other outcomes listed above.
- SAE of any grade assessed as related to study vaccine

In addition, any event, regardless of severity, which in the judgment of the PI represents a SAE, may be reported on an expedited basis.

An investigator will communicate an initial SAE report within 24 hours of site awareness of occurrence to the IND Sponsor by data entry in the database, which triggers an alert to the IND Sponsor MO. A written event summary by the investigator should be submitted to the IND Sponsor within 3 business days.

In order for the IND Sponsor to comply with regulations mandating sponsor notification of specified SAEs to the FDA within 7 and/or 15 calendar days, the investigator must submit additional information as soon as it is available.

5.4 IND Sponsor Reporting to the FDA

The IND Sponsor is responsible for making the determination of which SAEs are “serious and unexpected suspected adverse reactions” (SUSARs) as defined in 21 CFR 312.32.

- *Suspected adverse reaction* means any AE for which there is a reasonable possibility that the drug caused the AE.
- *Unexpected adverse event* means an AE that is not listed in the IB or is not listed at the specificity or severity that has been observed.

All SUSARs (as determined by the IND Sponsor) will be reported to the FDA as IND Safety Report per 21 CFR.32 as soon as possible but not exceeding 7 calendar days for unexpected fatal or life-threatening events and not exceeding 15 calendar days for other qualifying events. IND Safety Reports will be provided to the IRB.

The IND Sponsor will also submit an IND Annual Report of the progress of the investigation to the FDA as defined in 21 CFR 312.33.

5.5 Reporting to the IRB

The following information is consistent with NIH IRB Policy 801: Reporting Research Events, Version 1.0, effective July 1, 2019.

Reportable Event – An event that occurs during the course of human subjects research that requires notification to the IRB.

For the purposes of this policy, reportable events include the following:

- Unanticipated problems (UPs) involving risks to subjects or others
- Non-compliance (including major protocol deviations and noncompliance that is not related to a protocol deviation)
- Deaths related or possibly related to research activities
- New information that might affect the willingness of subjects to enroll or continue participation in the study.

5.5.1 Unanticipated Problem

An unanticipated problem (UP) is defined as any incident, experience, or outcome that meets **all** of the following criteria:

- **Unexpected** (in terms of nature, severity, or frequency) given (a) the research procedures that are described in the protocol, related documents, such as the IRB approved research protocol and informed consent document; and (b) the characteristics of the subject population being studied; **and**
- **Related or possibly related to** participation in the **research** (possibly related means there is a reasonable possibility that the incident, experience, or outcome may have been caused by the procedures involved in the research); **and**
- Suggests that the research **places subjects or others** (which may include research staff, family members or other individuals not directly participating in the research) **at a greater risk of harm** (including physical, psychological, economic, or social harm) related to the research than was previously known or expected.

A UP must be reported within 7 calendar days of an investigator becoming aware of the actual or suspected UP.

5.5.2 Non-Compliance

Non-compliance is the failure of investigator(s) to follow the applicable laws, regulations, or institutional policies governing the protection of human subjects in research, or the requirements or determinations of the IRB, whether intentional or not.

Non-compliance may be unintentional (e.g. due to lack of understanding, knowledge, or commitment), or intentional (e.g. due to deliberate choice to ignore or compromise the requirements of any applicable regulation, organizational policy, or determination of the IRB).

Non-compliance is further characterized as serious or continuing as follows:

- Serious non-compliance – Non-compliance, whether intentional or not, that results in harm or otherwise materially compromises the rights, welfare and/or safety of the subject. Non-compliance that materially effects the scientific integrity or validity of the research may be considered serious non-compliance, even if it does not result in direct harm to research subjects.

- Continuing non-compliance – A pattern of recurring non-compliance that either has resulted, or, if continued, may result in harm to subjects or otherwise materially compromise the rights, welfare and/or safety of subjects, affect the scientific integrity of the study or validity of the results. The pattern may comprise repetition of the same non-compliant action(s), or different noncompliant events.

Any actual or suspected non-compliance by any investigator or entity associated with the protocol must be reported to the IRB by the PI/designee within 7 calendar days of any investigator or individual associated with the protocol first becoming aware.

5.5.3 Protocol Deviation

A Protocol Deviation is a non-compliance defined as any change, divergence, or departure from the IRB-approved research protocol and are further characterized as major and minor as follows:

- Major Deviations – Deviations from the IRB approved protocol that have, or may have the potential to, negatively impact, the rights, welfare or safety of the subject, or to substantially negatively impact the scientific integrity or validity of the study.
- Minor Deviations – Deviations that do not have the potential to negatively impact the rights, safety, or welfare of subjects or others, or the scientific integrity or validity of the study.

For the reporting purposes, failure of subjects to comply with the research protocol does not represent non-compliance unless that failure is due to an action or omission of a member of the research team, for example, the failure to give adequate instruction to the subject.

A major deviation must be reported within 7 calendar days of an investigator becoming aware of an actual or suspected deviation. Although protocol deviations are also non-compliance, these should only be reported once as deviations. Major deviations resulting in death must be reported within 24 hours of the occurrence of the event or of any member of the study team becoming aware of the death.

Researchers are responsible for monitoring their studies throughout the year for adherence to the IRB approved protocol. The purpose of this monitoring is to identify major deviations and to look for trends in minor deviations that may indicate a systemic issue in how the study is being conducted that could potentially negatively impact the rights, safety, or welfare of participants or the study's ability to produce scientifically valid results. A series of minor deviations pointing toward a more global issue that could affect the rights, safety or welfare of the participant or affect the validity of the study should be reported as a major deviation. In all other instances, a summary of minor deviations should be provided to the IRB at the time of continuing review.

5.5.4 Death

Any death of a research subject that is possibly, probably or definitely related to the research must be reported within 24 hours of an investigator becoming aware of the death.

5.5.5 New Information

New information that might affect the willingness of a subject to enroll or remain in the study should be reported within 7 calendar days of an investigator first becoming aware.

5.5.6 Suspension or Termination of Research Activities

Any suspension or termination of research activities, including holds on new enrollment, placed upon the research by the study sponsor, NIH or NIH Institute/Center (IC) leadership, or any regulatory agency must be reported within 7 calendar days of an investigator becoming aware.

5.5.7 Expedited Reporting to the IRB

Death related to research must be reported within **24 hours.**

The following will be reported within **7 calendar days** of investigator awareness:

- Actual or suspected UPs;
- Actual or suspected non-compliance;
- Actual or suspected Major Protocol Deviations;
- SAEs that are actual or suspected UPs;
- New information that might affect the willingness of a subject to enroll or remain in the study;
- Suspension or termination of research activities, including holds on new enrollment, placed upon the research by the study sponsor, NIH or IC leadership, or any regulatory agency.

5.5.8 Annual Reporting to the IRB

The following will be reported to the IRB in summary at the time of Continuing Review:

- Summary of UPs and non-compliance;
- AEs, including SAEs that are not UPs, as a narrative summary statement indicating whether these events were within the expected range;
- Minor Protocol Deviations (aggregate summary);
- Any trends or events which in the opinion of the investigator should be reported.

6. STATISTICAL CONSIDERATIONS

6.1 Overview

This a dose escalation study to evaluate the safety and immunogenicity of a 3-dose regimen with the Trimer 4571 vaccine given with adjuvant. The primary objective is to evaluate the safety and tolerability of the vaccine administered with alum.

6.2 Objectives

The primary objectives are to evaluate the safety, tolerability of VRC-HIVRGP096-00-VP with alum. Reactogenicity will be closely monitored for 7 days after each product administration and safety evaluated by clinical visits through the study duration.

The immunogenicity of VRC-HIVRGP096-00-VP, administered with alum by either SC or IM route, is also of interest and will be summarized descriptively in this small sample.

6.3 Endpoints

6.3.1 Primary Endpoints: Safety

Assessment of product safety will include clinical observation and monitoring of hematological and chemical parameters. Reactogenicity will be closely monitored for 7 days after each injection and safety evaluated by clinical visits throughout the 40 weeks. See **Section 4.2** and **Appendix II** for details and specified time points.

The following parameters will be assessed for all study groups:

- Occurrence of solicited local reactogenicity symptoms beginning 7 days following each injection, through resolution of symptoms.
- Occurrence of solicited systemic reactogenicity symptoms for beginning 7 days following each injection, through the resolution of symptoms.
- Change from baseline in safety laboratory measures
- Occurrence of AEs of all severities
- Occurrence of SAEs or new chronic medical conditions that require ongoing medical management at any time throughout the study.

6.3.2 Secondary Endpoints: Immunogenicity

The principal immunogenicity endpoint is two weeks after the third dose as measured by ELICA.

6.3.3 Exploratory Endpoints: Immunogenicity

Cellular and humoral responses at specific timepoints after initiation of product administration for each group will be summarized.

6.4 Sample Size and Accrual

Recruitment will target 16 healthy adult subjects 18 to 50 years of age.

6.4.1 Power Calculations for Safety

The goal of the safety evaluation for this study is to identify safety concerns associated with injections of the investigational vaccine. Primary sample size calculations for safety are expressed in terms of ability to detect serious adverse experiences. Other sample size calculations for comparing the vaccination groups on adverse experiences are similar to the calculations for immunogenicity.

Table 2 describes the probability of observing 0 and 2 or more AEs in the 16 subjects, or the 3 or 5 subjects in group, based on a range of true underlying probabilities. The ability of the study to identify SAEs will be expressed in terms of the probability of observing a certain number of SAEs. If the rate of AEs is 1% or less there is a 95% chance, we will not see any in a group of 5 subjects and an 85% chance we will not see any in the full cohort of 16 subjects. If the rate is 30% or higher, there is a 97% chance we will see two or more subjects with an AE out of the full cohort of 16, but only a 47% chance we will see 2 or more in a group of 5.

Probabilities of observing 0 or more than 1 SAE are presented in **Table 2** for a range of possible true event rates. These calculations provide a complete picture of the severity of the sensitivity of this study design to identify potential safety problems with the vaccine.

Table 2: Probability of Observing Adverse Events (n=12)

True Event Rate	Pr (0/3)	Pr (2+/3)	Pr (0/5)	Pr (2+/5)	Pr (0/16)	Pr (2+/16)
0.01	0.97	<0.01	0.95	<0.01	0.85	<0.01
0.05	0.86	<0.01	0.77	0.02	0.44	0.19
0.10	0.73	0.03	0.59	0.08	0.19	0.49
0.20	0.51	0.10	0.33	0.26	0.03	0.86
0.30	0.34	0.21	0.17	0.47	<0.01	0.97

Table 3 gives the upper and lower bounds for 95% exact binomial confidence intervals of the true SAE rate at all possible numbers of events in a group of size 3 or 5, or in the full cohort of 16. If none of the 16 subjects experience SAEs, the 95% exact 2-sided upper confidence bound for the SAE rate is 21%. Note that the results for 9 or more events out of the full cohort of 16 are not explicitly listed, but by symmetry can be calculated as 1- the listed Rate and 95% CI for the number of non-events. For example, if there are 9 events out of 16, the rate would be 1-0.58=0.42 and the 95% CI would be 1-(0.20, 0.70)=(0.30, 0.80).

Table 3: 95% Confidence Intervals for the True Rate at All Possible Observed Rates

Outcome	Rate (95% CI)	Outcome	Rate (95% CI)	Outcome	Rate (95% CI)
0/3	0 (0, 0.71)	0/5	0 (0, 0.52)	0/16	0 (0, 0.21)
1/3	0.33 (<0.01, 0.91)	1/5	0.20 (<0.01, 0.72)	1/16	0.08 (<0.01, 0.30)
2/3	0.67 (0.09, 0.99)	2/5	0.40 (0.05, 0.85)	2/16	0.17 (0.02, 0.38)
3/3	1.00 (0.29, 1.00)	3/5	0.60 (0.15, 0.95)	3/16	0.25 (0.04, 0.46)
		4/5	0.80 (0.28, 0.99)	4/16	0.33 (0.07, 0.52)
		5/5	1.00 (0.48, 1)	5/16	0.42 (0.11, 0.59)
				6/16	0.50 (0.15, 0.65)
				7/16	0.58 (0.20, 0.70)
				8/16	0.67 (0.25, 0.75)

6.5 Statistical Analysis

Since enrollment is concurrent with receiving the first study injection, the expectation is that all subjects will receive at least one injection and therefore will provide some safety data. All statistical analyses will be performed using statistical software R. No formal multiple comparison adjustments will be employed for safety endpoints or immunogenicity endpoints.

6.5.1 Baseline Demographics

Baseline characteristics including demographics and laboratory measurements will be summarized using descriptive statistics.

6.5.2 Safety Analysis

Reactogenicity: The number and percentage of subjects experiencing each type of reactogenicity sign or symptom will be tabulated by severity. For a given sign or symptom, each subject's reactogenicity will be counted once under the maximum severity for all assessments.

Adverse Events: AEs will be coded into Medical Dictionary for Regulatory Activities (MedDRA) preferred terms. The number and percentage of subjects experiencing each specific AE will be tabulated by severity and relationship to study product. For the calculations in these tables, each subject's AE will be counted once under the maximum severity or strongest recorded causal relationship to treatment.

A complete listing of AEs for each subject will provide details including severity, relationship to treatment, onset, duration and outcome.

Safety Laboratory Values: Boxplots of local laboratory values will be generated for baseline values and for values measured during the course of the study. Each boxplot will show the 1st quartile, the median, and the 3rd quartile. Outliers, or values outside the boxplot, will also be plotted. If appropriate, horizontal lines representing boundaries for abnormal values will be plotted.

6.5.3 Analysis of Immune Responses

The primary immunogenicity time point is 2 weeks after the third dose, and will be summarized by responses both within subjects (compared to baseline) and between subjects (between dose cohorts or route cohorts). All available data will be analyzed from subjects who receive at least one dose.

6.5.4 Missing Data

Missing responses will be assumed to be missing completely at random; excluding data that are missing completely at random is not expected to bias the analyses. Analysis will include all samples available at each study time point.

6.5.5 Randomization of Treatment Assignments

At the start of the study, subjects will be randomized 1:1 to Groups 1 and 2. If the criteria for the first dose escalation are met and procedures for the interim safety review are completed then subsequent subjects will be randomized 1:1 into Groups 3 and 4. The subject and the study clinicians will be informed of the subject's group assignment upon completing enrollment in the database.

If subjects accrued to a study group do not complete the number of injections and/or follow-up duration specified, then additional subjects may be accrued in that group.

7. STUDY PRODUCTS AND PHARMACY PROCEDURES

7.1 Study Products

The study products were manufactured for the VRC by the VRC Production Plant, operated under contract by the Vaccine Clinical Materials Program (VCMP), Leidos Biochemical, Inc., Frederick, MD, according to current Good Manufacturing Practice (cGMP) regulations and must meet lot release specifications prior to clinical use. This study includes one investigational vaccine, one adjuvant and one diluent as follows:

- VRC-HIVRGP096-00-VP (Trimer 4571) drug product is a sterile, aqueous, buffered solution filled under aseptic conditions into single dose vials at a concentration of 500 mcg/mL. The formulation buffer is comprised of 10 mM Sodium Phosphate, 10 mM Sodium Chloride, 7.5% Sorbitol, 0.01% PF-68 at pH 7.2. The drug product is aseptically filled at a volume of 1.2 ± 0.10 mL in 3 mL glass vials. Vials contain a clear, colorless solution; no turbidity, some small white or translucent particles may be present.
- Aluminum Hydroxide Suspension, alum, adjuvant is composed of Alhydrogel® 2% (Brenntag Biosector, Frederikssund Denmark) diluted with water for injection to a concentration of 5 mg/mL. Adjuvant is aseptically filled at a volume of 0.7 ± 0.1 mL.
- VRC-PBSPLA043-00-VP, diluent, is comprised of Phosphate Buffered Saline (PBS) aseptically filled into single dose vials at a volume of 1.2 ± 0.10 mL.

7.2 Study Product Labels

Vials of study product will be individually labeled with the name of the material, number, volume, lot number, concentration, storage instructions, Investigational Use Statement ("Limited by Federal Law to Investigational Use") and manufacturer information.

7.3 Study Product Storage

VRC-HIVRGP096-00-VP (Trimer 4571): The product will be shipped within the recommended temperature range using appropriate shipping configurations, to the study pharmacist or designee. The product label designates the long-term storage temperature as -35°C to -15°C. Clinical site storage in a qualified, continuously monitored, temperature-controlled freezer with a temperature range of -45°C to -10°C is acceptable.

Vials should not be refrozen after thaw. Thawed vials can be stored at 2°C to 8°C for up to 48 hours and/or at 15°C to 27°C for up to 24 hours.

Aluminum Hydroxide Suspension (alum adjuvant): Vials of adjuvant are stored until use in a qualified, continuously monitored, temperature-controlled refrigerator with a temperature range of 2°C to 8°C and should not be frozen.

VRC-PBSPLA043-00-VP (diluent): Vials of diluent (PBS) are stored until use in a qualified, continuously monitored, temperature-controlled freezer between -45°C to -10°C. Vials of diluent should not be refrozen after thawing.

7.3.1 Temperature Excursions

The site pharmacist must promptly report any storage temperature excursions outside of the normal allowance for the storage device to the PI and IND Sponsor (**Appendix I**). The affected product must be quarantined in a separate area. If the excursion results in thawed material, DO NOT REFREEZE; store the thawed, vialed material at 2°C to 8°C.

Provide the following information regarding the excursion to the IND Sponsor: lot number, fill volume and number of vials affected; temperature range and length of excursion, including data log reports; handling of materials post excursion (e.g. transfer to alternate storage, including times); visual inspection data of the materials (e.g. did the materials appear to have remained frozen, were vials cracked, etc.). The IND Sponsor will notify the site pharmacist if continued clinical use of the product is acceptable or not.

7.4 Preparation of Study Product-Adjuvant for IM and SC Administration

Refer to the group assignment for the study subject. The pharmacy will label the syringe before delivery to the clinic with the subject identifier, the date, and the expiration time for administration.

Preparation of the adjuvanted product must be done by a Pharmacist or designee in a clean preparation unit with limited access under a laminar flow hood to maintain sterile conditions.

The following preparation instructions apply:

7.4.1 Preparation of the 500 mcg Dose with Adjuvant

1. Thaw 1 vial of VRC-HIVRGP096-00-VP at controlled room temperature (15°C to 27°C) for about 30 minutes. Vials should not be moved directly from the freezer to a refrigerator to thaw.
2. Swirl the thawed, equilibrated VRC-HIVRGP096-00-VP vial for about 30 seconds with sufficient force to mix the solution yet avoiding foaming. DO NOT SHAKE THE VIALS. If some white to translucent particles are observed, vials may be used for preparation of the SC and/or IM administration. Remove 1 vial of alum adjuvant from 2°C to 8°C storage and equilibrate at controlled room temperature (15°C to 27°C) for a minimum of 15 minutes.
3. Mix the vial of alum adjuvant by gently inverting 5 times.
4. Add 0.12 mL of alum adjuvant directly to a thawed vial of VRC-HIVRGP096-00-VP (1.2 mL vaccine; total preparation volume = 1.32 mL).
5. Gently invert the combined vial 5 times to mix, incubate at controlled room temperature (15°C to 27°C) for about 15 minutes.
6. Invert vial gently 5 times to mix, then withdraw 1.1 mL into a new syringe for administration.
7. If not administered immediately, invert syringe 5 times to mix immediately prior to administration. Refer to Section 4.2.3 for storage information prior to administration.

7.4.2 Preparation of the 100 mcg Dose with Adjuvant

1. Thaw 1 vial of VRC-HIVRGP096-00-VP and 1 vial of VRC-PBSPLA043-00-VP at controlled room temperature (15ºC to 27ºC)for about 30 minutes. Vials should not be moved directly from the freezer to a refrigerator to thaw.

   Swirl the thawed, equilibrated VRC-HIVRGP096-00-VP vial for about 30 seconds with sufficient force to mix the solution yet avoiding foaming. DO NOT SHAKE THE VIALS. If some white to translucent particles are observed, vials may be used for the preparation of the SC and/or IM administration.
2. Remove 1 vial of alum adjuvant from 2ºC to 8ºC storage and equilibrate at controlled room temperature (15ºC to 27ºC) for a minimum of 15 minutes.
3. Mix the vial of alum adjuvant by gently inverting 5 times.
4. Transfer 0.30 mL (150 mcg) of VRCHIVRGP096-00-VP into a new sterile vial using a syringe.
5. Transfer 0.15 mL of alum adjuvant into the same vial.
6. Transfer 1.05 mL of VRC-PBSPLA043-00-VP into the same vial (total preparation volume =1.5 mL).
7. Gently invert vials 5 times to mix, then incubate at controlled room temperature (15ºC to 27ºC) for about 15 minutes.
8. Invert vial gently 5 times to mix, then withdraw 1.0 mL (100 mcg) into a new syringe for administration.
9. If not administered immediately, invert syringe gently 5 times to mix immediately prior to administration. Refer to Section 4.2.3 for storage information prior to administration.

7.5 Study Product Accountability

7.5.1 Documentation

The study pharmacist or designee will be responsible for maintaining an accurate record of the study group codes, inventory, and an accountability record of study product supplies. Electronic documentation as well as paper copies may be used.

7.5.2 Disposition

Empty vials and the unused portion of the vial should be discarded in a biohazard containment bag for incineration or decontamination by autoclave and disposed in accordance with institutional or pharmacy policy. Partially used vials will not be administered to other subjects or used for *in vitro* experimental studies. Any unopened vials that remain at the end of study will be discarded at the discretion of the Sponsor in accordance with policies that apply to investigational agents.

8. HUMAN SUBJECT PROTECTIONS AND ETHICAL OBLIGATIONS

This research study will be conducted in compliance with the protocol, Good Clinical Practice (GCP) regulations, and all applicable regulatory requirements.

8.1 Institutional review board

The protocol, proposed informed consent form, and any proposed advertising material will be submitted to the IRB for review and approval. The PI is responsible for obtaining annual IRB approval/renewal throughout the duration of the study.

8.2 Subject recruitment and enrollment

All study activities will be carried out at the NIH CC. Study subjects will be recruited through on-site and off-site advertising done for the screening protocol, VRC 500 (11-I-0164). Effort will be made to include women and minorities in proportions similar to that of the community from which they are recruited.

8.2.1 Participation of Children

Children are not eligible to participate in this clinical trial because the study product has not been previously evaluated in adults. If the product is assessed as safe and immunogenic, other protocols designed for children may be conducted in the future.

8.2.2 Participation of Site Employees

NIH employees and members of their immediate families may participate in this protocol. VRC will follow the Guidelines for the Inclusion of Employees in NIH Research Studies and will give each employee a copy of the "NIH Information Sheet on Employee Research Participation" and a copy of the "Leave Policy for NIH Employees Participating in NIH Medical Research studies."

Neither participation nor refusal to participate will have an effect, either beneficial or adverse, on the subject's employment or work situation. The NIH information sheet regarding NIH employee research participation or work situation. The NIH information sheet regarding NIH employee participation will be distributed to all potential subjects who are NIH employees. The employee subject's privacy and confidentiality will be preserved in accordance with NIH CC and NIAID policies. For NIH employee subjects, consent will be obtained by an individual who is independent of the employee's team. If the individual obtaining consent is a co-worker to the subject, independent monitoring of the consent process will be included through the Bioethics Consultation Service. Study staff will be trained on obtaining potentially sensitive and private information from co-workers or subordinates.

8.3 Informed Consent

The study informed consent form (ICF) describes the investigational products and all aspects involved in protocol participation. Before a subject's participation in the study, it is the investigator's responsibility to obtain written informed consent from the subject after adequate explanation of the aims, methods, anticipated benefits, and potential hazards of the study and before any protocol-specific procedures are conducted or study agent is administered. The Assessment of Understanding will be completed before the study consent is signed.

The acquisition of informed consent will be documented in the subject's medical record, as required by 21 CFR 312.62. The informed consent form will be signed and personally dated by the subject and by the person who conducted the informed consent discussion. The original signed informed consent form will be retained in the medical record and a copy will be provided to the subject.

8.4 Subject confidentiality

The investigator must ensure that the subject's anonymity is maintained and will ensure that no information identifying the subject will be released to any unauthorized party. Subjects will not be identified in any reports of this study. All records will be kept confidential to the extent provided by federal, state and local law. Medical records will be made available for review when required by authorized agencies and regulatory authorities only under the guidelines set by the U.S. Federal Privacy Act and by relevant country-specific regulatory authorities. Direct access includes examining, analyzing, verifying, and reproducing any records and reports that are important to the evaluation of the study. The investigator is obligated to inform the subjects that the above-named representatives will review their study-related records without violating the confidentiality of the subjects. Stored study research samples are labeled by a code that only the study team can link to the subject. The requirement to maintain subject confidentiality is included in the ICF.

8.5 Risks and Benefits

8.5.1 Risks

Risks of the Trimer 4571 vaccine: This is the first study in humans of the Trimer 4571 vaccine. The risks noted are based on risks of vaccines in general.

Potential side effects resulting from intramuscular and subcutaneous injections include stinging, discomfort, redness of skin, or mild bruising at vaccine injection site. As with any injection procedure, infection at the site of injection is a possible risk. Signs of infection at the injection site include: severe pain, redness, swelling, warmth or drainage. Subjects may exhibit general signs and symptoms associated with administration of the vaccine injection, including fever, chills, rash, aches and pains, nausea, dizziness and fatigue. These side effects will be monitored, but are generally short term, mild to moderate severity and usually do not require treatment.

There may be side effects from the study products, which may be serious or life threatening that we do not know about yet.

This vaccine is intended to generate antibodies, which may cause a positive HIV antibody result in standard diagnostic tests. A positive or indeterminate test may have a negative employment and social impact. HIV PCR will be used to exclude or confirm HIV infection. HIV-1 and HIV-2 Antibody Confirmation and Differentiation results that are indeterminate or positive may be vaccine-induced.

Risks of Aluminum Hydroxide Suspension: Aluminum is the most common adjuvant used in human vaccines licensed by the FDA used in billions of individuals over decades of clinical use [35] [29]. Side effects are generally limited to minor local reactions at the injection site [29]. Other more severe local reactions like erythema, SC nodules, contact hypersensitivity and granulomatous inflammation may occur [33].

Risks of Blood Drawing: Blood drawing may cause pain, bruising, fainting, and, rarely, infection at the site where the blood is taken.

Risks during Pregnancy: We do not know the possible effects of the study vaccine on the fetus or nursing infant. Therefore, women and adolescents of reproductive potential will be tested for pregnancy prior to administration of each dose of study product. Women will be asked to notify the site immediately if they suspect or learn they are pregnant during this study. In case of pregnancy, subjects will continue to be followed for safety. The subject will be contacted about the outcome of a pregnancy that begins during the study.

Other Risks: It is possible that the standard medical tests performed as part of this research protocol will result in new diagnoses. Depending on the medical findings and consequences of being provided with the new medical information about health status, the study subject may view this aspect of study participation as either a risk or a benefit. Any such information will be shared and discussed with the subject and, if requested by the subject, may be forwarded to the subject's primary health care provider for further workup and management.

8.5.2 Benefits

Study subjects will not receive direct health benefit from study participation. This protocol is not designed to provide treatment for any condition. Others may benefit from knowledge gained in this study that may aid in the development of an HIV vaccine. The investigational vaccine is not expected to provide protection from HIV infection.

8.6 Plan for Use and Storage of Biological Samples

To be eligible for this protocol, subjects must be willing to allow stored specimens to be used in the future for studying infectious diseases, immune function, vaccine responses, and other medical conditions. In general, testing performed at a research laboratory is not for diagnostic purposes and results will not be available to the study subject. If tests show evidence of any acute or chronic condition, subjects will be informed of the results and advised to seek appropriate medical care for the condition.

8.6.1 Use of Samples, Specimens and Data

Samples, specimens and data collected under this protocol may be used to conduct protocol-related safety and immune response evaluations, exploratory laboratory evaluations related to the type of infection the study product was designed to prevent, exploratory laboratory evaluations related to vaccine or infectious disease research in general and for research assay validation.

Genetic testing may be performed in accordance with the genetic testing information that was included in the study ICF. HLA testing may be done in association with identifying factors linked with the immune response development or progression of infections.

8.6.2 Storage and Tracking of Blood Samples and Other Specimens

All of the stored research samples are labeled by a code that only the site can link to the subject. Samples are stored at secure facilities with limited access including the VIP formerly VITL, Gaithersburg, MD and VRC Laboratories, Building 40, Bethesda, MD or other approved CRO facilities. Data will be kept in password-protected computers. Only investigators or designees will have access to the samples and data. Samples will be tracked in the Laboratory Information Management System (LIMS) database or using another software designed for this purpose (e.g., Freezerworks, GlobalTrace).

8.6.3 Disposition of Samples, Specimens and Data at Completion of the Protocol

In the future, other investigators (both at NIH and outside) may wish to study these samples and/or data. Regulatory approval through the proper human subjects protection agency will be sought prior to any sharing of samples that constitute human subjects research. The research use of stored, unlinked or unidentified samples may be exempt from the need for IRB review and approval. When appropriate exemption may be obtained through the proper regulatory procedures.

At the time of protocol termination, samples will remain in the VIP facility or VRC laboratories or, after IRB approval, be transferred to another repository. Regulatory oversight of the stored samples and data may be transferred to a stored samples protocol as part of the IRB-approved termination plan. Data will be archived by the VRC in compliance with requirements for retention of research records, or after the IRB and the IND Sponsor approval, it may be either destroyed or transferred to another repository.

8.6.4 Loss or Destruction of Samples, Specimens or Data

Any loss or unanticipated destruction of samples (for example, due to freezer malfunction) or data (for example, misplacing a printout of data with identifiers) that compromises the scientific integrity of the study will be reported to the IRB in accordance with institutional policies. The PI will also notify the IRB if the decision is made to destroy the remaining samples.

8.7 Compensation

Compensation for time and inconvenience of study participation will be provided to subjects in accordance with the standards for compensation of the NIH Clinical Research Volunteer Program. The compensation per visit will be $275 for injection visits and $175 for visits that include a blood draw only. Compensation for any clinic visit that does not include a blood draw will be $75 and compensation for completion of electronic diary card will be $25. The total compensation for the subject is based on the number of study clinic visits and injections completed. The approximate total compensation is $2825.

Subjects will receive compensation about 2 weeks after each completed visit by direct deposit. Compensation may need to be reported to the Internal Revenue Service (IRS) as taxable income

8.8 Safety Monitoring

8.8.1 Protocol Safety Review Team (PSRT)

Close cooperation between the designated members of the Protocol Team will occur to evaluate and respond to individual AEs in a timely manner. The study clinicians will conduct a daily safety review of any new clinical data. The PSRT is comprised of the Study Chair, PI(s), Associate Investigators, Study Coordinator, Protocol Specialists, other Study Clinicians and other clinicians. The PSRT will review the summary study safety data reports on a weekly basis through 4 weeks after the last subject receives the final study injection in order to be certain that the vaccine has an acceptable safety profile and will continue to monitor the study safety data reports on a monthly basis through completion of the last study visit.

9. ADMINISTRATIVE AND LEGAL OBLIGATIONS

9.1 Protocol Amendments and Study Termination

Protocol amendments must be made only with the prior approval of the IND Sponsor. Agreement from the investigator must be obtained for all protocol and ICF amendments. All amendments will be submitted to the IRB for approval.

The VRC, NIAID, NIH, FDA, and other regulatory authorities reserve the right to terminate the study. The PI will notify the IRB in writing of the study's completion or early termination and provide documentation to the IND Sponsor.

9.2 Study Documentation and Study Records Retention

The PI will maintain a list of appropriately qualified persons to whom trial duties have been delegated.

Source documents are original documents, data, and records from which the subject's data are obtained. These include but are not limited to hospital records, clinical and office charts, laboratory and pharmacy records, and correspondence. Long term storage of source documents may be in the form of electronic files.

The PI and staff are responsible for maintaining a comprehensive and centralized filing system of all study-related (essential) documentation, suitable for inspection at any time by representatives from the IND Sponsor, VRC, NIAID, NIH, IRB, FDA, and/or applicable regulatory authorities. Elements include:

- Subject files containing completed ICF and supporting copies of source documentation,
- Study files containing the protocol with all amendments, the IB, and copies of all correspondence with the IRB

In addition, all original source documentation must be maintained and be readily available.

All essential documentation should be retained by the institution for the same period of time required for medical records retention. The FDA requires study records to be retained for up to two years after marketing approval or refusal (21 CFR 312.62). No study document should be destroyed without prior written agreement between the IND Sponsor and the PI.

9.3 Data Collection, Data Sharing, and Protocol Monitoring

9.3.1 Data Collection

Clinical research data will be collected in a secure electronic web-based clinical data management system (CDMS) through a contract research organization, The Emmes Company, LLC (Rockville, MD). Extracted data without subject identifiers will be sent to the PSRT for safety review and to protocol statistician for statistical analysis as needed.

9.3.2 Data Sharing

Data generated in this study will be shared as de-identified data in the government-funded public repository, www.ClinicalTrials.gov. Data may be shared prior to publication at approved public

presentations or for collaborative development and will be shared at the time of publication and no later than 1 year after the primary completion date.

9.3.3 Source Documents

The site will maintain appropriate medical and research records for this trial, in compliance with ICH-GCP, regulatory and institutional requirements for the protection of confidentiality of subjects. Source data are all information, original records of clinical findings, observations, or other activities in a clinical trial necessary for the reconstruction and the evaluation of the trial. Examples of these original documents and data records include, but are not limited to, medical records, paper diary cards, laboratory reports, pharmacy records and other research records maintained for the clinical trial.

9.3.4 Protocol Monitoring

The IND Sponsor or their authorized representatives are responsible for ensuring integrity of study data and compliance with the protocol. The PI will allow the study monitors, the IRB, the FDA, and other applicable regulatory authority representatives to inspect study documents (e.g., consent forms, drug distribution forms, and case report forms) and pertinent hospital or clinic records for confirmation of the study data. Site visits by study monitors will be made to monitor the following: study operations, the quality of data collected in the research records, the accuracy and timelines of data entered in the database, and to determine that all process and regulatory requirements are met. Study monitoring visits will occur as defined by the IND Sponsor approved monitoring plan.

9.4 Language

All written information and other material to be used by subjects and investigative staff must use vocabulary and language that are readily understood.

9.5 Policy Regarding Research-Related Injuries

The study site will provide short-term medical care for any injury resulting from participation in this research. In general, the NIH, the Clinical Center, or the U.S. Federal Government will provide no long-term medical care or financial compensation for research-related injuries.

10. REFERENCES

1. Jaffe, H.W., D.J. Bregman, and R.M. Selik, *Acquired immune deficiency syndrome in the United States: the first 1,000 cases.* J Infect Dis, 1983. **148**(2): p. 339-45.
2. Fauci, A.S., *The acquired immune deficiency syndrome. The ever-broadening clinical spectrum.* Jama, 1983. **249**(17): p. 2375-6.
3. Gottlieb, M.S., *Pneumocystis pneumonia--Los Angeles. 1981.* Am J Public Health, 2006. **96**(6): p. 980-1; discussion 982-3.
4. *Kaposi's sarcoma and Pneumocystis pneumonia among homosexual men--New York City and California.* MMWR Morb Mortal Wkly Rep, 1981. **30**(25): p. 305-8.
5. Barre-Sinoussi, F., et al., *Isolation of a T-lymphotropic retrovirus from a patient at risk for acquired immune deficiency syndrome (AIDS).* Science, 1983. **220**(4599): p. 868-71.
6. Gallo, R.C., et al., *Isolation of human T-cell leukemia virus in acquired immune deficiency syndrome (AIDS).* Science, 1983. **220**(4599): p. 865-7.
7. *Human T-cell leukemia virus infection in patients with acquired immune deficiency syndrome: preliminary observations.* MMWR Morb Mortal Wkly Rep, 1983. **32**(18): p. 233-4.
8. UNAIDS. *Fact sheet: 2017 statistics*. 2017; Available from: www.unaids.org/sites/default/files/media_asset/UNAIDS_FactSheet_en.pdf.
9. Anderson, R.M. and G.F. Medley, *Epidemiology of HIV infection and AIDS: incubation and infectious periods, survival and vertical transmission.* Aids, 1988. **2 Suppl 1**: p. S57-63.
10. Veugelers, P.J., et al., *Determinants of HIV disease progression among homosexual men registered in the Tricontinental Seroconverter Study.* Am J Epidemiol, 1994. **140**(8): p. 747-58.
11. Schmit, J.C. and B. Weber, *Recent advances in antiretroviral therapy and HIV infection monitoring.* Intervirology, 1997. **40**(5-6): p. 304-21.
12. Ippolito, G., et al., *The changing picture of the HIV/AIDS epidemic.* Ann N Y Acad Sci, 2001. **946**: p. 1-12.
13. Esparza, J., *A brief history of the global effort to develop a preventive HIV vaccine.* Vaccine, 2013. **31**(35): p. 3502-18.
14. Hallenberger, S., et al., *Inhibition of furin-mediated cleavage activation of HIV-1 glycoprotein gp160.* Nature, 1992. **360**(6402): p. 358-61.
15. Hallenberger, S., et al., *The role of eukaryotic subtilisin-like endoproteases for the activation of human immunodeficiency virus glycoproteins in natural host cells.* J Virol, 1997. **71**(2): p. 1036-45.
16. Wyatt, R. and J. Sodroski, *The HIV-1 envelope glycoproteins: fusogens, antigens, and immunogens.* Science, 1998. **280**(5371): p. 1884-8.
17. Eckert, D.M. and P.S. Kim, *Mechanisms of viral membrane fusion and its inhibition.* Annu Rev Biochem, 2001. **70**: p. 777-810.
18. Mascola, J.R. and D.C. Montefiori, *The role of antibodies in HIV vaccines.* Annu Rev Immunol, 2010. **28**: p. 413-44.
19. Pancera, M., et al., *Structure and immune recognition of trimeric pre-fusion HIV-1 Env.* Nature, 2014. **514**(7523): p. 455-61.
20. Munro, J.B., et al., *Conformational dynamics of single HIV-1 envelope trimers on the surface of native virions.* Science, 2014. **346**(6210): p. 759-63.

21. Kwon, Y.D., et al., *Crystal structure, conformational fixation and entry-related interactions of mature ligand-free HIV-1 Env.* Nat Struct Mol Biol, 2015. **22**(7): p. 522-31.
22. Guttman, M., et al., *Antibody potency relates to the ability to recognize the closed, pre-fusion form of HIV Env.* Nat Commun, 2015. **6**: p. 6144.
23. Sanders, R.W., et al., *A next-generation cleaved, soluble HIV-1 Env trimer, BG505 SOSIP.664 gp140, expresses multiple epitopes for broadly neutralizing but not non-neutralizing antibodies.* PLoS Pathog, 2013. **9**(9): p. e1003618.
24. Chuang, G.Y., et al., *Structure-Based Design of a Soluble Prefusion-Closed HIV-1 Env Trimer with Reduced CD4 Affinity and Improved Immunogenicity.* J Virol, 2017. **91**(10).
25. Brewer, J.M., *(How) do aluminium adjuvants work?* Immunol Lett, 2006. **102**(1): p. 10-5.
26. Kuroda, E., C. Coban, and K.J. Ishii, *Particulate adjuvant and innate immunity: past achievements, present findings, and future prospects.* Int Rev Immunol, 2013. **32**(2): p. 209-20.
27. FDA. *Common Ingredients in U.S. Licensed Vaccines*. 2014; Available from: http://www.fda.gov/biologicsbloodvaccines/safetyavailability/vaccinesafety/ucm187810.htm
28. He, P., Y. Zou, and Z. Hu, *Advances in aluminum hydroxide-based adjuvant research and its mechanism.* Hum Vaccin Immunother, 2015. **11**(2): p. 477-88.
29. Brito, L.A., P. Malyala, and D.T. O'Hagan, *Vaccine adjuvant formulations: a pharmaceutical perspective.* Semin Immunol, 2013. **25**(2): p. 130-45.
30. Brito, L.A. and D.T. O'Hagan, *Designing and building the next generation of improved vaccine adjuvants.* J Control Release, 2014. **190**: p. 563-79.
31. Oleszycka, E. and E.C. Lavelle, *Immunomodulatory properties of the vaccine adjuvant alum.* Curr Opin Immunol, 2014. **28**: p. 1-5.
32. Schijns, V.E. and E.C. Lavelle, *Trends in vaccine adjuvants.* Expert Rev Vaccines, 2011. **10**(4): p. 539-50.
33. Baylor, N.W., W. Egan, and P. Richman, *Aluminum salts in vaccines--US perspective.* Vaccine, 2002. **20 Suppl 3**: p. S18-23.
34. Clements, C.J. and E. Griffiths, *The global impact of vaccines containing aluminium adjuvants.* Vaccine, 2002. **20 Suppl 3**: p. S24-33.
35. Petrovsky, N. and J.C. Aguilar, *Vaccine adjuvants: current state and future trends.* Immunol Cell Biol, 2004. **82**(5): p. 488-96.
36. Gupta, R.K., et al., *Adjuvant properties of aluminum and calcium compounds.* Pharm Biotechnol, 1995. **6**: p. 229-48.
37. Roldao, A., et al., *Virus-like particles in vaccine development.* Expert Rev Vaccines, 2010. **9**(10): p. 1149-76.

APPENDIX I: CONTACT INFORMATION

APPENDIX II: SCHEDULE OF EVALUATIONS

VRC 018 Schedule of Evaluations																			
**Visit Number**		**01**	**02**	**02A**	**03**	**04**	**05**	**06**	**06A**	**07**	**08**	**09**	**10**	**10A**	**11**	**12**	**13**	**14**	**15**
**Study Week (Day after injection)**			W0	(Day 1)	W1	W2	W4	W8	(Day 1)	W9	W10	W12	W20	(Day 1)	W21	W22	W24	W32	W40
**Study Day**		**-56 to 0**	0	1	7	14	28	56	57	63	70	84	140	141	147	154	168	224	280
**Study Procedures**	**Tube***	**[1]Screen**																	
Assessment of Understanding			X																
Informed Consent			X																
[2] Physical Exam		X	X		X	X	X	X		X	X	X	X		X	X	X	X	X
[3] Medical History		X	X		X	X	X	X		X	X	X	X		X	X	X	X	X
[4] Concomitant Medications		X	X		X	X	X	X		X	X	X	X		X	X	X	X	X
[5] Study Injection			**X**					**X**					**X**						
[6] Reactogenicity Diary			X					X					X						
Phone Contact				X					X					X					
[7] Pregnancy Prevention		X	X					X					X						
[8] HIV Risk Reduction Counseling		X	X			X					X					X		X	X
**Clinical Labs**																			
CBC with differential	EDTA	3	3		3	3		3		3	3		3		3	3			
ALT, Creatinine	GLT	4	4		4	4		4		4	4		4		4	4			
[7] Pregnancy Test (urine or serum)		[X]	[X]					[X]					[X]						4
HIV Ab/Ag Combo	EDTA	3				3					3					3		3	3
HIV-1 RNA Quantification	EDTA										6					6		6	6
[9] HLA Typing	EDTA											20							
**Research Samples**																			
Serum	SST	16	16		8	8	8	8		16	8	8	8		16	16	16	8	16
PBMC and Plasma	EDTA	40	80		10	10	10	10		40	10	10	10		40	40	40	10	40
**Daily Volume (mL)**		**66**	**103**	**0**	**25**	**28**	**18**	**25**	**0**	**63**	**34**	**38**	**25**	**0**	**63**	**72**	**56**	**27**	**69**
**Cumulative Volume (mL)**		**66**	**169**	**169**	**194**	**223**	**241**	**266**	**266**	**329**	**364**	**402**	**427**	**427**	**490**	**563**	**619**	**647**	**717**

Visit Windows:
Visits 02A, 06A, and 10A (+1 day)
Visits 03, 07, 11 (± 1 day)
Visits 04, 05, 08, 09, 12 (± 2 days)
Visits 6, 10, 13, 14, 15 (±7 days)

Target Dates:
Visits 02A - 06 are relative to Day 0
Visits 06A - 10 are relative to Visit 06
Visits 10A - 15 are relative to Visit 10

*Tube types for clinical labs are according to institutional requirements and are shown to estimate blood volumes. Different tubes for clinical evaluations may be used to meet site requirements. Research sample tube types and blood volumes must be used as shown or as otherwise instructed by the IND Sponsor.

[1] **Screening evaluations** at Visit 01 are performed under VRC 500, no more than 56 days before Day 0 and include clinical lab results for eligibility, complete physical exam (including height and weight) and medical history. Research labs drawn during screening that are not within 56 days of enrollment do not need to be repeated.

[2] **Physical exam** is performed on Day 0 and at each subsequent visit and includes blood pressure, temperature, pulse and respirations. On Day 0, physical exam will also include weight to determine BMI for eligibility. At other visits, a targeted physical exam will be performed as needed, based on subject report or indications of illness.

[3] **Medical history** A complete history is performed during screening. At enrollment and subsequently, an interim medical history may be performed.

[4] **Concomitant medications** are recorded during screening, at enrollment and at each subsequent visit as noted in Section 4.2.5.

[5] **Study Injection:** At least 30 minutes after each study injection and prior to clinic discharge, subjects will have vital signs taken and the injection site will be assessed. Subject who do not receive the 2nd and or 3rd study injection will continue study participation under the Modified Schedule below.

[6] **Reactogenicity diary:** Subjects will complete a **7-day diary** as noted in Section 4.2.4. If any solicited reactogenicity is unresolved by Day 7, it will continue to be reviewed with the subject at each visit until resolved.

[7] **Pregnancy prevention counseling and testing** is performed for women of childbearing potential as indicated above and at other visits if deemed clinically appropriate. A negative pregnancy test result must be confirmed for each woman of childbearing potential on the day of and prior to each study injection.

[8] **HIV risk reduction and post-test counseling**, including potential for VISP will be conducted as indicated above and at other visits if deemed clinically appropriate. A wallet card and letter regarding VISP will be provided to volunteers as noted in Section 4.4.

[9] **HLA typing** is shown at Visit 9 or 10 but may be completed at any timepoint during the study. ***If HLA typing was performed at the NIH CC within 2 years prior to Visit 10, it does not need to be repeated.*** HLA test results will be printed, placed in the research chart and entered into the database.

VRC 018 Schedule of Evaluations<br>Modified for Discontinuation of Study Injections(s)						
	**Visit Number**	**06**	**09**	**10**	**13**	**14**
	**Week of Study**	**W8**	**W12**	**W20**	**W24**	**W32**
	**Day of Study**	**56**	**84**	**140**	**168**	**224**
**Study Procedures**	**Tube***					
[1] Physical exam		X	X	X	X	X
[2] Medical history		X	X	X	X	X
[3] Concomitant Medications		X	X	X	X	X
[4] Pregnancy Prevention Counseling		X				
[5] HIV Risk Reduction Counseling		X		X	X	X
**Clinical Labs**						
CBC with differential	EDTA	3		3		
ALT, Creatinine	GLT	4		4		
[4] Pregnancy test: urine or serum		[X]			4	4
HIV Ab/Ag Combo	EDTA	3		3	3	3
HIV-1 RNA Quantification	EDTA	6		6	6	6
[6] HLA Typing	EDTA			20		
**Research Samples**						
Serum	SST	8	8	8	8	16
PBMC and Plasma	EDTA	10	10	10	10	40
**Daily Volume (mL)**		**34**	**18**	**54**	**31**	**69**
**Cumulative Volume (mL), 1 Study Injection**		**276**	**294**	**349**	**381**	
**Cumulative Volume (mL), 2 Study Injections**				**457**	**489**	**559**

If only 1 study injection given:

Follow original study schedule through Visit 05 then move to Modified Schedule for Visits 06, 09, 10, 13. Final study visit will be Visit 13.

If only 2 study injections given:

Follow original study schedule through Visit 09 then move to Modified Schedule for Visits 10, 13, 14. Final study visit will be Visit 14.

Visit Windows: all visits on modified schedule (±7 days)

*Tube types for clinical labs are according to institutional requirements and are shown to estimate blood volumes. Different tubes for clinical evaluations may be used to meet site requirements. Research sample tube types and blood volumes must be used as shown or as otherwise instructed by the IND Sponsor.[1]

Physical exam a targeted physical exam will be performed as needed, based on subject report or indications of illness.

[2] **Medical history** an interim medical history may be performed.

[3] **Concomitant medications** are recorded at each subsequent visit as noted in Section 4.2.5.

[4] **Pregnancy prevention counseling and testing** is performed for women of childbearing potential as indicated above and at other visits if deemed clinically appropriate. A negative pregnancy test result must be confirmed for each woman of childbearing potential on the day of and prior to each study injection.

[5] **HIV risk reduction and post-test counseling**, including potential for VISP will be conducted as indicated above and at other visits if deemed clinically appropriate. A wallet card and letter regarding VISP will be provided to volunteers as noted in Section 4.4.

[6] **HLA typing** is shown at Visit 9 or 10 but may be completed at any timepoint during the study. ***If HLA typing was performed at the NIH CC within 2 years prior to Visit 10, it does not need to be repeated.*** HLA test results will be printed, placed in the research chart and entered into the database.

APPENDIX III: ASSESSING RELATIONSHIP AND GRADING SEVERITY OF ADVERSE EVENTS

Assessment of Causality Relationship of an Adverse Event to Study Vaccine:

The relationship between an adverse event (AE) and the vaccine will be assessed by the investigator on the basis of his or her clinical judgment and the definitions below.

- **Definitely Related**. The AE and administration of study product are related in time, and a direct association can be demonstrated.
- **Probably Related**. The AE and administration of study product are reasonably related in time, and the AE is more likely explained by study product than other causes.
- **Possibly Related**. The AE and administration of study product are reasonably related in time, but the AE can be explained equally well by causes other than study product.
- **Not Related**. There is not a reasonable possibility that the AE is related to the study product.

For purposes of preparing data reports in which AE attributions are limited to "**Related**" or "**Not Related**", in this protocol, the "Definitely, Probably and Possibly" attributions will be mapped to the "Related" category. The definitions that apply when these two categories alone are used are as follows:

- **Related** – There is a reasonable possibility that the AE may be related to the study product.
- **Not Related** – There is not a reasonable possibility that the AE is related to the study product.

Table for Grading Severity of Adverse Events

U.S. Department of Health and Human Services, National Institutes of Health, National Institute of Allergy and Infectious Diseases, Division of AIDS. Division of AIDS (DAIDS) Table for Grading the Severity of Adult and Pediatric Adverse Events, Corrected Version 2.1 [July 2017]. Available from: https://rsc.tech-res.com/clinical-research-sites/safety-reporting/daids-grading-tables

The table will be used as posted at the link above with the following exemptions:

- Weight loss will be recorded as an adverse event only if it is considered deleterious to the subject's health.
- For severity grading of the solicited bruising parameter at the product administration site, the definitions based on size of the largest diameter and listed for the "Injection Site Erythema or Redness" will be used. The severity grade definition for "Bruising" provided under the Dermatologic Clinical Conditions will be used only for unsolicited adverse events involving bruising at other body locations.
- Creatinine changes will be graded on the basis of the upper limit of normal provided by the grading table and not change from baseline.
- Creatinine clearance changes will be graded according to ml/min provided by the grading table and not change from baseline.
- Subclinical CMP results for sodium, potassium, chloride, bicarbonate, BUN, and glucose will not be considered an AE unless grade 2 or greater.

INSTITUTE: National Institute of Allergy and Infectious Diseases

STUDY NUMBER: 19-I-0031 PRINCIPAL INVESTIGATOR: Martin Gaudinski, M.D.

STUDY TITLE: VRC 018: A Phase I Dose Escalation, Randomized, Open-Label Clinical Trial to Evaluate Dose, Safety, Tolerability and Immunogenicity of a HIV-1 Vaccine, VRC-HIVRGP096-00-VP, with Alum in Healthy Adults

Continuing Review Approved by the IRB on 09/30/19
Amendment Approved by the IRB on 09/30/19 (B) Date Posted to Web: 10/09/19
Standard, Version 3.0

INTRODUCTION

We invite you to take part in a research study at the National Institutes of Health (NIH).

First, we want you to know that:

> Taking part in NIH research is entirely voluntary.
>
> You may choose not to take part, or you may withdraw from the study at any time. In either case, you will not lose any benefits to which you are otherwise entitled. However, to receive care at the NIH, you must be taking part in a study or be under evaluation for study participation.
>
> You may receive no benefit from taking part. The research may give us knowledge that may help people in the future.

Second, some people have personal, religious or ethical beliefs that may limit the kinds of medical or research treatments they would want to receive (such as blood transfusions). If you have such beliefs, please discuss them with your NIH doctors or research team before you agree to the study.

Now we will describe this research study. Before you decide to take part, please take as much time as you need to ask any questions and discuss this study with anyone at NIH, or with family, friends or your personal physician or other health professional.

PURPOSE AND PLAN OF THE STUDY

This is a research study of an experimental vaccine against the human immunodeficiency virus (HIV) called Trimer 4571. “Experimental” means that the study vaccine has not been approved by the U.S. Food and Drug Administration (FDA). The FDA allows this vaccine to be used for research purposes only. This vaccine has never been given to humans before this study. We do not know if the vaccine works. The main purpose of this study is to see if the experimental vaccine is safe and if there are any side effects. We also want to study immune responses to the vaccine including cells that may recognize and fight HIV. There is currently no licensed vaccine to prevent HIV infection.

STUDY PRODUCT

The Trimer 4571 vaccine in this study was developed by the Vaccine Research Center (VRC) at the NIH. It was made at the VRC Pilot Plant in Frederick, MD. Vaccines are substances that are given to help the body fight off an infection. When you get a dose of this vaccine, it will look like a protein that is on the outside surface of HIV. Your body may

make an immune response (including antibodies) based on this protein. **This vaccine does not have any live or killed HIV in it. It is impossible for you to get HIV from this vaccine.**

You should not expect this experimental vaccine to protect you from HIV infection.

People in this study will get the Trimer 4571 vaccine mixed with another study product called an adjuvant. Adjuvants are substances that help make your body's immune response to the vaccine better. The adjuvant in this study will be aluminum hydroxide (alum). Alum has been used for over 60 years in billions of vaccinations with licensed vaccines and has been found to be safe. The use of alum as an adjuvant in this study has been reviewed and approved by the FDA.

ELIGIBILITY

You are eligible to participate in this study because you have completed the screening process and:

- You are 18-50 years of age,
- You agree not to become pregnant during this study,
- You have a physical exam and blood test results that meet study requirements,
- You do not have any serious medical problems as determined by your screening, and
- You agree to avoid behavior that will put you at risk for getting HIV.

STUDY PLAN

The study plan includes enrollment of about 16 people. You will have about 15 study visits and you will be in the study for about 9 months.

The study will have 4 different groups of about 3 to 5 people each. Groups 1 and 2 will get a low dose (100 mcg) and Groups 3 and 4 will get a high dose (500 mcg). We will start by enrolling people into the low dose groups. After we review that there are no safety concerns related to the low dose, we will enroll people to get the high dose.

The first 6 people in the study will be randomly assigned (like flipping a coin) to a group to get the low dose by either intramuscular (IM) or subcutaneous (SC) injection. The next 10 people will be randomly assigned to get the high dose by either IM or SC injection. Once on study, you will know which group you are in.

The 4 possible groups are shown below:

**VRC 018 Vaccination Schedule**						
**Group**	**Subjects**	**Route**	**Dose**	**Day 0**	**Week 8**	**Week 20**
1	3	IM	Low	X	X	X
2	3	SC	Low	X	X	X
3	5	IM	High	X	X	X
4	5	SC	High	X	X	X
**Total**	16	IM = intramuscular injection; vaccination into the muscle<br>SC = subcutaneous injection; vaccination under the skin into the tissue<br>Alum will be mixed with Trimer 4571 for all groups				

STUDY PROCEDURES

If you agree to take part in this study, you will get a total of three injections as shown in the table above. The injections will be given either IM into the upper arm muscle or SC under the skin of your upper arm. We will watch you in the clinic for at least 30 minutes after the injection. Each of the three vaccination visits will last about 4 to 6 hours.

If you are a woman who is able to get pregnant, we will do a pregnancy test before you get each injection. If the test shows that you are pregnant, you will not get the injection. Counseling related to pregnancy prevention and HIV risk-reduction will be performed throughout the study.

We will give you a thermometer and ask you to check your temperature every day for 7 days after each injection. You will need to record your highest temperature and any symptoms you may have. We will also give you a device to measure any redness or swelling at the injection site. Even if you do not feel sick, it is very important that you record this information. We will give you a password to a secure website to enter this information on an electronic form or diary. If you do not have a computer, you may use a paper diary instead. The clinic staff will call you the day after each injection to check on you. We will also be available to you by phone 24 hours a day so you can report any concerning side effects.

If you feel sick at any time during the study, you should contact the clinic right away. We may ask you to come to the clinic for an examination before your next planned visit. It is very important that you follow the instructions we give you.

Follow-up clinic visits will last about 1 to 2 hours. At each visit, we will check you for any health changes or problems. We will check you for possible side effects from the vaccine. We will ask how you are feeling and if you have taken any medications. At each clinic visit, we will draw about 2-12 tubes of blood from you for clinical tests and for research, depending on the visit. If any of your test results show a health problem, we will tell you about it as soon as possible. You might need to have extra clinic visits and laboratory tests if you have health changes that we need to check.

Experimental vaccine studies follow a set schedule. The study schedule for visits allows some flexibility, but it is important that you work with the staff to follow the schedule. You should try to not miss any visits. You should contact the clinic staff as soon as possible if you need to change the date or time of any study visit.

MONITORING OF THE STUDY

A group of physicians and scientists at NIH will monitor this study. This group will review the information from the study and will pay close attention to any reactions. If serious side effects occur, injections may be delayed or canceled.

GENETIC TESTING

Some of the blood drawn from you as part of this study will be used for genetic tests. Some genetic tests are done in research studies to see if different types of immune response are related to genetic differences in people. Your blood sample used in these genetic tests will not have your name on it and the results will not be in your medical record. These tests are not used to check your health and we will not tell you the results.

A special genetic test called HLA typing may be done by the NIH Clinical Center medical laboratory. These results will be in your medical record, but they will not be used to check your health. If HLA typing is done in a research laboratory, the result will not be in your medical record. Any genetic testing, including HLA typing is for research purposes only. Any genetic information collected or learned about you will be kept confidential. Medical records, including HLA test results, are kept securely. We will not give any genetic information that is in your medical record to anyone without your permission.

STORED SAMPLES

We will collect blood samples from you during the study. We will keep these samples to study your immune responses to the vaccine and for future research to learn more about vaccines, the immune system, and/or other medical conditions. Results from research done from your stored samples are not for medical care and will not be in your medical record or reported to you.

Labeling of Stored Samples: Your stored samples will be labeled by a code (like a number) that only the study team can link to you. Any information that could identify you, like your name or date of birth, will kept confidential as much as allowable by law. Even with these protections, there is a small chance that information identifying you will be accidently given to someone who should not get it.

Future Studies: In the future, other investigators (at NIH or outside of NIH) may wish to study your stored samples. When your stored samples are shared, they will be marked with a code. Your samples will not have any identifying information on them. Some information about you, such as your gender, age, health history, or ethnicity may also be shared with other researchers.

Any future research studies using your samples will be conducted in a way that protects the rights and privacy of study participants.

Your stored samples will be used only for research and will not be sold. The research done with your materials may be used to develop new products in the future, but you will not receive payment for such products.

HUMAN DATA SHARING

To advance science, it is helpful for researchers to share information they get from studying humans by putting it into shared scientific databases. Researchers can then study the information combined from many studies to learn even more about health and diseases.

If you agree to take part in this study, some of your data will be placed into one or more scientific databases. We will remove identifying information like your name, address, and birth date. The data may then be used for future research and shared broadly for research purposes. Only researchers who are approved to access the database may be able to see and use your information. You will not get any direct benefits from future research that uses your data and information.

You may stop participating in this study at any time and withdraw permission for your individual data, specimens, and health information to be used for additional or future research. You may ask to have your research data destroyed. However, it may not be possible to withdraw or delete data once they have been shared with other researchers.

POSSIBLE STUDY RISKS

Possible risks of injections: Temporary stinging, pain, redness, soreness, itchiness, swelling, or bruising, and rarely infection.

Possible risks of blood drawing: Pain, bleeding, feeling lightheaded, or fainting, and rarely, infection at the site where the blood is taken.

Possible risks of any vaccine: Fever, chills, rash, aches and pains, nausea, headache, dizziness, and feeling tired or unwell. Some people have allergic reactions to vaccines. These types of reactions are usually greatest within the

first 24 hours after an injection and typically last 1 to 3 days. Over-the-counter medicine, such as acetaminophen, may be used to help relieve these symptoms.

As with any medicine, including vaccines, there is a chance of reactions. These reactions are usually mild and go away on their own, but serious reactions are also possible. A serious allergic reaction with symptoms like hives, trouble breathing, or sudden weakness may happen shortly after any vaccination. This is called "anaphylaxis" and may be life-threatening. While you are waiting in the clinic after the injections, we will monitor you for anaphylaxis. Treatment for anaphylaxis will be given right away if it happens.

Possible risks of the Trimer 4571 vaccine: This is the first study to give the Trimer 4571 vaccine to humans. There may be side effects, even serious or life-threatening ones, which we do not know about yet. Subjects may exhibit general signs and symptoms associated with a vaccine injection, including fever, chills, rash, aches and pains, nausea, dizziness and fatigue. These side effects do not last long and usually do not require treatment.

We will tell you if we learn about any important new findings or serious side effects during the study that may change your mind about your desire to continue in the study.

Possible risks of alum: Alum is the most common adjuvant used in human vaccines licensed by the FDA. In healthy subjects, side effects are generally mild local reactions at the injection site that may include tenderness, redness, and/or swelling. Another more severe local reaction at the injection site may include lumps under the skin.

Possible risks during pregnancy: We do not know if getting the study products will affect a fetus. Therefore, women who can get pregnant must agree to use an effective method of birth control starting at least 21 days before getting the first injection and during the 40 weeks of study participation. We will discuss effective birth control methods with you. If you are pregnant or want to become pregnant during the study, you cannot participate.

During the study, if you think you might be pregnant, you must tell the clinic staff right away. If you are pregnant, you will not get any more vaccinations. You will be asked to continue with some follow-up visits so that we can check on your health. We will ask you about the outcome of the pregnancy

Possible risks of genetic testing: Unplanned release of information that could be used by insurers or employers to discriminate against you or your family; discovering a gene that suggests risk of disease for you or your family; discovering unknown family relationships.

Possible risks of data sharing: Information in the shared databases could be linked back to you and used to discriminate against you or your family. State and federal laws provide some protections against genetic and pre-existing conditions discrimination.

Possible risk of a positive result on an HIV test caused by the vaccine: At the time of enrollment, we will test you for HIV using an HIV antibody test. Your results must be negative to be on the study.

After you get the study vaccine, it is very likely that your body will make antibodies to HIV, even though you do not have HIV infection. This is called a vaccine-induced seropositive (VISP) test result. The antibodies or VISP cannot be passed to your sexual partner.

If someone thinks you have HIV infection because of VISP even though you do not, you could have difficulties. You could face discrimination with disability insurance, long-term care insurance, or employment.

During the study we will test your blood for HIV antibodies and also use a test that looks for the actual virus. This makes it possible for us to tell the difference between VISP and actual HIV infection. To avoid any confusion, you should not get tested for HIV outside of the VRC Clinic during the study.

If you do have HIV antibodies from the experimental vaccinations, we do not know how long they will last in your

blood. They could last for several months, several years, or possibly for life.

You may not be able to donate blood while you have VISP.

If you have VISP after you leave the study, you may get tested for HIV at the VRC Clinic or the NIH for as long as you need it, at no cost to you. We will give you a wallet card that tells you who to contact at any time during or after the study if you need help with issues arising from VISP. We will also give you a letter of explanation about VISP that you can share with your healthcare provider or others.

Unknown risks: We do not know if the study product will affect how you respond to any HIV infection or HIV vaccine that you may get in the future.

POSSIBLE BENEFITS

This study is not designed to benefit you or to protect you from HIV infection. You and others may benefit in the future from the information that we learn from the study.

COST OF PARTICIPATIION

There are no costs to you for taking part in this study. You or your health insurance will have to pay for all medical costs for medical care that you get outside this study. It is possible that you may have some costs that are not covered by the study compensation we give you.

COMPENSATION TO YOU FOR YOUR PARTICIPATION

You will get compensation for the time and inconvenience of study participation.

The compensation is: $175 for scheduled visits that include blood drawing, $275 for vaccination visits, $25 for finishing all 7 days of each electronic diary card, and $75 for clinic visits that do not include blood drawing. Total compensation for completion of the study and all injections is estimated to be $2,825. Actual compensation is based on the number and type of visits you complete.

You will get your compensation about 2 weeks after each completed visit by direct deposit into a bank account that you specify to the volunteer payment office. Compensation may need to be reported to the Internal Revenue Service (IRS) as taxable income.

REASONS FOR STOPPING YOUR STUDY INJECTIONS

You may not get all of your planned study vaccinations. Reasons may include:

- You get a serious illness that needs ongoing medical care.
- You have a serious side effect thought to be due to the study vaccine.
- You enroll in another research study at the same time you are in this study.
- You become pregnant.
- You need to get treatment with a medication that affects your immune system.
- The study is stopped by regulatory agencies, the study sponsor or study investigators. If this happens, we will tell you why.

REASONS FOR REMOVING YOU FROM THE STUDY

You may be taken out of the study without your consent. Reasons for this may include:

- Continuing in the study could hurt you,
- You don't follow instructions or keep your appointments, or
- The study is stopped by the NIH, FDA or other regulatory authorities

If you get the first injection but do not complete the schedule, we will ask that you continue with all of your planned follow-up visits until the end of the study. It is important that we continue to check your health even if you do not get the second or third dose.

VOLUNTARY PARTICIPATION

Your participation in this study is completely voluntary. You can choose to stop taking part in the study at any time. There is no penalty or loss of benefits for choosing to leave the study.

ALTERNATIVES

This study is not designed to treat or prevent any disease. You may choose to not take part in this study.

CONFLICT OF INTEREST

The research staff is checked yearly for conflicts of interest. You may ask the research team for more information. This study may have investigators who are not NIH employees. Non-NIH investigators are expected to follow the principles of the Protocol Review Guide.

The NIH, including some members of the VRC scientific staff, developed the investigational vaccine being used in this research study. The results of this study could play a role in whether the FDA will approve the vaccine for sale at some time in the future. If approved, the future sale of the vaccine could lead to payments to NIH and some NIH scientists. By U.S. law, government scientists are required to receive such payments for their inventions. You will not get any money from the development or sale of the product.

CLINICALTRIALS.GOV

A description of this clinical trial will be available on www.ClinicalTrials.gov. This website will not include information that can identify you. At most, the website will include a summary of the results. You can search this website at any time.

OTHER PERTINENT INFORMATION

1. Confidentiality. When results of an NIH research study are reported in medical journals or at scientific meetings, the people who take part are not named and identified. In most cases, the NIH will not release any information about your research involvement without your written permission. However, if you sign a release of information form, for example, for an insurance company, the NIH will give the insurance company information from your medical record. This information might affect (either favorably or unfavorably) the willingness of the insurance company to sell you insurance.

The Federal Privacy Act protects the confidentiality of your NIH medical records. However, you should know that the Act allows release of some information from your medical record without your permission, for example, if it is required by the Food and Drug Administration (FDA), members of Congress, law enforcement officials, or authorized hospital accreditation organizations.

2. Policy Regarding Research-Related Injuries. The Clinical Center will provide short-term medical care for any injury resulting from your participation in research here. In general, no long-term medical care or financial compensation for research-related injuries will be provided by the National Institutes of Health, the Clinical Center, or the Federal Government. However, you have the right to pursue legal remedy if you believe that your injury justifies such action.

3. Payments. The amount of payment to research volunteers is guided by the National Institutes of Health policies. In general, patients are not paid for taking part in research studies at the National Institutes of Health. Reimbursement of travel and subsistence will be offered consistent with NIH guidelines.

4. Problems or Questions. If you have any problems or questions about this study, or about your rights as a research participant, or about any research-related injury, contact the Principal Investigator, Martin Gaudinski, MD or the Study Coordinator, Laura Novik, MA, BSN, RN [redacted]
You may also call the Clinical Center Patient Representative at [redacted].

5. Consent Document. Please keep a copy of this document in case you want to read it again.

COMPLETE APPROPRIATE ITEM(S) BELOW:

Adult Study Subject's Consent

I have read the explanation about this study and have been given the opportunity to discuss it and to ask questions. I hereby consent to take part in this study.

____________________________________ ______
Signature of Adult Participant/Legal Representative Date

____
Print Name

THIS CONSENT DOCUMENT HAS BEEN APPROVED FOR USE FROM SEPTEMBER 30, 2019 THROUGH SEPTEMBER 29, 2020.

____________________________________ ______ ____________________________________ ______
Signature of Investigator/Person Obtaining Consent Date Signature of Witness Date

____________________________________ ____________________________________
Print Name Print Name
